# Carbon Nanodots-Based Sensors: A Promising Tool for Detecting and Monitoring Toxic Compounds

**DOI:** 10.3390/nano15100725

**Published:** 2025-05-11

**Authors:** Duyen H. H. Nguyen, Arjun Muthu, Tamer Elsakhawy, Mohamed H. Sheta, Neama Abdalla, Hassan El-Ramady, József Prokisch

**Affiliations:** 1 Institute of Animal Science, Faculty of Agricultural and Food Sciences and Environmental Management, Biotechnology and Nature Conservation, University of Debrecen, 138 Böszörményi Street, 4032 Debrecen, Hungary; joe.prokisch@gmail.com; 2 Institute of Life Sciences, Vietnam Academy of Science and Technology, 9/621 Vo Nguyen Giap Street, Linh Trung Ward, Thu Duc City, Ho Chi Minh City 70000, Vietnam; 3 Doctoral School of Nutrition and Food Science, University of Debrecen, 4032 Debrecen, Hungary; arjunvmuthu@gmail.com; 4 Institute of Agricultural Chemistry and Soil Science, Faculty of Agricultural and Food Sciences and Environmental Management, University of Debrecen, 138 Böszörményi Street, 4032 Debrecen, Hungary; 5 Agricultural Microbiology Research Department, Soils, Water and Environment Research Institute (SWERI), Agricultural Research Center (ARC), Giza 12619, Egypt; drelsakhawyg@gmail.com; 6 Soils and Water Department, Faculty of Agriculture, Al-Azhar University, Cairo 11884, Egypt; mohamedsheta.205@azhar.edu.eg; 7 Plant Biotechnology Department, Biotechnology Research Institute, National Research Centre, 33 El Buhouth St., Dokki, Giza 12622, Egypt; neama_ncr@yahoo.com; 8 Soil and Water Department, Faculty of Agriculture, Kafrelsheikh University, Kafr El-Sheikh 33516, Egypt

**Keywords:** rapid detection, carbon dots, probes, heavy metal, contaminants, ultrasensitive sensor devices

## Abstract

The increasing prevalence of toxic compounds in food, agriculture, and the environment presents a critical challenge to public health and ecological sustainability. Carbon nanodots (CNDs), with their excellent photoluminescence, biocompatibility, and ease of functionalization, have emerged as highly promising materials for developing advanced sensors that target hazardous substances. This review provides a comprehensive overview of the synthesis, functionalization, and sensing mechanisms of CND-based sensors, highlighting their versatile application in detecting toxic compounds such as heavy metals, pesticides, mycotoxins, and emerging contaminants. The article outlines recent advancements in fluorescence, electrochemical, and colorimetric detection strategies and presents key case studies that illustrate the successful application of CNDs in real-world monitoring scenarios. Furthermore, it addresses the challenges associated with reproducibility, scalability, selectivity, and sensor stability and explores future directions for integrating CNDs with smart and sustainable technologies. This review emphasizes the transformative potential of CNDs in achieving rapid, cost-effective, and environmentally friendly toxin detection solutions across multiple domains.

## 1. Introduction

The widespread presence of toxic compounds in food, agricultural products, and the environment has emerged as a critical global concern. These contaminants present risks to human health and contribute to economic losses. For example, nutrient pollution in freshwater bodies across the United States leads to annual losses of at least USD 4 billion, mainly due to decreases in lakefront property values and recreational usage [1]. Between 1975 and 2020, the global economic impact of aquatic and semi-aquatic invasive plants surpassed USD 32 billion. The majority of these costs were attributed to damages within freshwater ecosystems [2]. Effective monitoring and mitigation strategies are urgently needed to address the widespread impact of toxic compounds on ecosystems and economies. These toxins—including pesticides, heavy metals, mycotoxins, and industrial pollutants—not only threaten human health but also compromise food safety, environmental integrity, and agricultural productivity [3,4]. While agrochemicals have significantly increased food production, their residues impact terrestrial and aquatic ecosystems, including coastal marine systems [5].

The need for environmental monitoring stems from several critical concerns. Exposure to pollutants like heavy metals and synthetic organic compounds has been linked to severe health effects, including neurotoxicity, endocrine disruption, and an increased risk of cancer [6,7]. Pollutants also disrupt biodiversity, harming aquatic organisms, terrestrial wildlife, and plant life, leading to long-term ecological imbalances [8,9]. Maintaining water quality is critical for safe drinking water, agricultural irrigation, and industrial processes. Reliable detection systems help enforce environmental safety standards and prevent contamination-related hazards [10,11].

Regulatory bodies worldwide mandate pollution monitoring to enforce environmental safety standards, highlighting the need for reliable detection techniques [12]. Early pollutant detection enables timely intervention, reducing the risk of large-scale contamination [13]. Accurate monitoring data also play a key role in public awareness, informed decision making, and sustainable environmental management [14].

Carbon nanodots (CNDs) have gained attention for their diverse sensing applications due to their water solubility, low toxicity, biocompatibility, and tunable photoluminescence [15,16]. These nanomaterials can be customized through heteroatom doping, composite formation, and metal complexation to enhance selectivity and sensitivity toward specific toxins [15]. CND-based sensors offer advantages such as simplicity, affordability, and high detection accuracy [15], making them strong candidates for biosensing, drug delivery, bioimaging, and environmental monitoring [17].

This review explores advancements in CND-based sensors for detecting toxic compounds in food, agriculture, and the environment. It discusses synthesis and functionalization strategies, outlines key sensing mechanisms, and highlights practical applications. Additionally, challenges related to stability, reproducibility, and selectivity are examined, with future directions aimed at integrating CNDs into next-generation environmental monitoring systems.

## 2. Fundamentals of Carbon Nanodots (CNDs) in Sensing

Carbon nanodots (CNDs) are carbon-based nanomaterials, typically under 10 nm in size, characterized by water solubility, tunable photoluminescence, and biocompatibility [18,19]. Structurally, they possess a carbon core with various surface functional groups and exhibit either crystalline or amorphous forms depending on the synthesis method [18]. CNDs can be synthesized via top–down or bottom–up approaches using methods like arc discharge, laser ablation, hydrothermal, microwave-assisted techniques, and pyrolysis [20,21]. The most commonly used synthesis methods for CNDs are presented in Table 1, including their advantages, limitations, and key references. Their optical and electronic properties—such as strong photoluminescence, high stability, and excitation-dependent emission—make CNDs highly suitable for diverse applications, including biosensing, bioimaging, drug delivery, nano-farming, and energy conversion [19,20,22]. These properties also contribute to their eco-friendliness and cost effectiveness, enhancing their appeal across disciplines.

**Figure 1:**
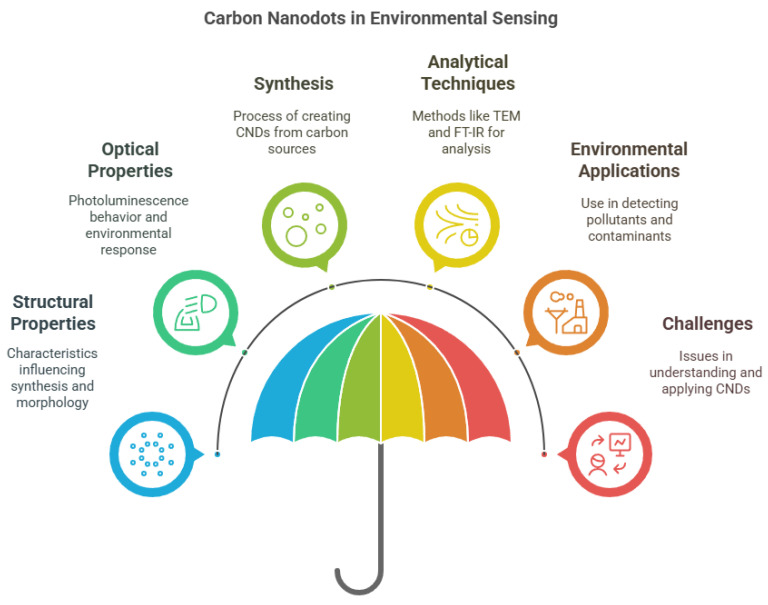
Primary aspects of carbon nanodots (CNDs) for sensing applications.

The structural and functional characteristics of CNDs are crucial to their sensing performance (Figure 1). Their fluorescence and electrochemiluminescence properties are particularly valuable in detecting contaminants and environmental changes [16]. Characterization tools like TEM, FT-IR, and XPS are used to evaluate their morphology and surface chemistry [23]. Functionalization or doping of CNDs further enhances their selectivity and sensitivity toward specific analytes [24]. For instance, incorporating molecularly imprinted polymers (MIPs) creates hybrid materials capable of targeting specific pollutants with improved reliability [25]. Their fluorescence behavior, influenced by solvent polarity and environmental factors, enables real-time chemical sensing [23].

Due to these unique attributes, CNDs have become a preferred platform for fluorescence-based sensing systems. The integration of CNDs with MIPs has further refined detection capabilities in complex matrices [25]. Despite their promise, continued research is needed to fully understand and optimize the structural mechanisms influencing their performance and long-term stability in real-world conditions.

**Table 1:** Comparative overview of carbon nanodot (CND) synthesis methods.

Method	Advantages	Limitations	References
Arc discharge	Produces high-quality nanostructures with fewer defects Simple and cost-effective equipment setup	Difficult to control size distribution and purity High temperatures can limit material types	[26]
Laser ablation	Generates high-purity nanoparticles with controlled size and shape Suitable for various nanocarbons	High energy input required Limited scalability due to small laser-irradiating area	[26,27]
Hydrothermal	Ability to synthesize substances unstable at melting point Produces large, high-quality crystals	Requires expensive autoclaves Inability to observe crystal growth in steel vessels	[28]
Microwave assisted	Rapid and energy-efficient synthesis Facilitates surface modification and defect engineering	Potential for uneven heating Limited to materials that can absorb microwave radiation	[29]
Pyrolysis	Simple and scalable process Applicable to a wide range of carbon-rich precursors	Requires high temperatures Risk of aggregation or structural collapse if poorly controlled	[30]

## 3. Carbon Nanodot-Based Sensors: Design and Mechanisms

CNDs have garnered significant attention due to their unique optical and physicochemical properties, including strong photoluminescence, excellent biocompatibility, low toxicity, and ease of surface functionalization, which make them highly suitable for a wide range of sensing applications in food safety, environmental monitoring, and biomedicine [31,32].

### 3.1. Sensor Design and Synthesis

CND-based sensors are developed using various synthesis methods, such as hydrothermal, solvothermal, and microwave-assisted techniques [33]. The properties of the resulting CNDs—including their size, surface chemistry, and emission wavelength—are highly tunable depending on the precursors and synthesis conditions [34]. These sensors are typically designed to detect analytes through changes in fluorescence, electrochemical signals, or colorimetric properties upon interaction with the target compound [35,36].

To enhance selectivity and performance, CNDs can be functionalized with molecularly imprinted polymers (MIPs), aptamers, or antibodies, which improve the binding affinity and specificity for target pollutants [37].

### 3.2. Fluorescence-Based Detection Mechanisms

The most common detection strategy involves changes in the fluorescence behavior of CNDs upon interaction with analytes. This includes both fluorescence quenching (turn-off sensors) and fluorescence enhancement (turn-on sensors).

#### Fluorescence Quenching Mechanisms

Fluorescence quenching occurs when the fluorescence intensity of CNDs is reduced due to their interaction with specific analytes. Various mechanisms explain this phenomenon:

- Static quenching: This involves the formation of non-fluorescent ground-state complexes between CNDs and analytes, often leading to altered absorption spectra. This type of quenching is sensitive to temperature changes [38]. For instance, ref. [39] used static quenching in the detection of chlortetracycline with nitrogen-doped CNDs.
- Dynamic quenching: This happens when excited-state CNDs collide with quencher molecules, transferring energy or electrons. It affects the fluorescence lifetime but not the absorption spectrum. Increasing the temperature enhances this quenching type [40,41]. Researchers applied dynamic quenching to detect malachite green in food matrices.
- Förster resonance energy transfer (FRET): In FRET-based systems, energy transfers from an excited donor (CNDs) to a nearby acceptor within ~10 nm. This leads to decreased donor fluorescence and enhanced acceptor emission. The presence of an analyte can reverse quenching by displacing the quencher and restoring CND fluorescence [42,43]. Ref. [44] demonstrated this mechanism for detecting Aflatoxin B1. Additionally, bicolor fluorescent molecular sensors offer promising capabilities for detecting cations through various mechanisms, including intramolecular charge transfer, excimer/exciplex formation, and FRET [45] to sense ultralow hazardous elemental traces [46].
- Inner filter effect (IFE): An IFE occurs when excitation or emission light is absorbed by another species in the system. This requires overlap between the absorber’s absorption spectrum and the CND’s excitation/emission wavelengths [47]. Refs. [48,49] used IFE to detect tinidazole in milk using N-doped CNDs.
- Photoinduced electron transfer (PET): PET involves electron transfer between CNDs and an analyte after photoexcitation, influencing the fluorescence output [50]. This often accompanies or overlaps with other quenching mechanisms.

### 3.3. Electrochemical Detection

CNDs possess excellent redox properties and can be integrated into electrochemical sensors. These systems detect changes in current or potential as CNDs interact with analytes. Incorporating conductive materials like graphene or metal nanoparticles enhances the sensitivity [36]. This approach is particularly useful for detecting pesticides, heavy metals, and pharmaceuticals.

### 3.4. Colorimetric Detection

CND-based colorimetric sensors rely on visible color changes upon analyte interaction, often due to the following:

- Aggregation-induced changes: Target analytes cause CND aggregation, altering their optical properties.
- Enzyme-mimicking activity: CNDs can mimic peroxidase activity, catalyzing oxidation reactions that produce colorimetric signals (e.g., in H_2_O_2_ or pesticide detection).

### 3.5. Comparison with Other Carbon Nanomaterials

Compared to other carbon-based nanomaterials like graphene quantum dots (GQDs), carbon nanotubes (CNTs), and carbon nanofibers (CNFs), CNDs offer unique advantages in sensing:

- GQDs: Although they provide high sensitivity, their synthesis is more complex and less scalable [51].
- CNTs: These are known for their high conductivity and strength, so they are better suited for electrochemical sensing but raise environmental and health concerns due to their fibrous morphology [52].
- CNFs: CNFs have a high aspect ratio and provide good mechanical properties, similar to CNTs, making them useful for various sensor applications, particularly in electrochemical devices. However, unlike CNDs, CNFs generally lack significant photoluminescence, which limits their use in optical sensors. CNFs also require energy-intensive synthesis methods, which can be a disadvantage in terms of cost effectiveness and scalability [53].

CNDs strike a balance between safety, tunability, and optical performance, making them ideal for a broad range of optical and electrochemical sensing platforms [54,55].

## 4. Toxic Compounds in Food, Agriculture, and the Environment

### 4.1. Heavy Metal Contaminants

Toxic compounds such as heavy metals pose serious threats to ecosystems, human health, and agriculture [56]. Originating from industrial discharge, mining, wastewater irrigation, and agrochemical overuse, heavy metals persist in the environment and agriculture, leading to bioaccumulation in crops and health risks through the food chain [57,58,59]. These chemicals can persist and accumulate over time, leading to long-term contamination that impacts not only the local agricultural areas but also distant ecosystems through runoff and atmospheric deposition [60]. Identifying heavy metals is essential due to their profound environmental and health effects. Metals like lead, mercury, arsenic, cadmium, chromium, copper, and nickel can cause serious health problems [61]. Exposure to these metals can result in both acute and chronic conditions, including neurological disorders, developmental issues, and cancers [62]. Heavy metals do not degrade and can accumulate in the environment, leading to soil and water pollution. This pollution can harm plants, animals, and aquatic life, disrupting ecosystems and biodiversity [63]. Heavy metals can build up in living organisms and become more concentrated up the food chain. Even low environmental concentrations can become highly concentrated in top predators, including humans, posing significant health risks [64]. These pollutants alter soil pH, reduce microbial diversity, and impair nutrient cycling, ultimately degrading soil fertility [58,65,66]. In plants, metals like Pb, Cd, and As cause chlorosis, stunted growth, and diminished yields, threatening food security [57]. Early detection of heavy metals enables timely intervention and remediation [67], preventing further contamination and reducing long-term health and environmental impacts [68]. Carbon nanodots (CNDs) have emerged as a promising tool for this purpose due to their unique properties, such as high photoluminescence, biocompatibility, and ease of functionalization [69].

### 4.2. Organic Pollutants

**Figure 2:**
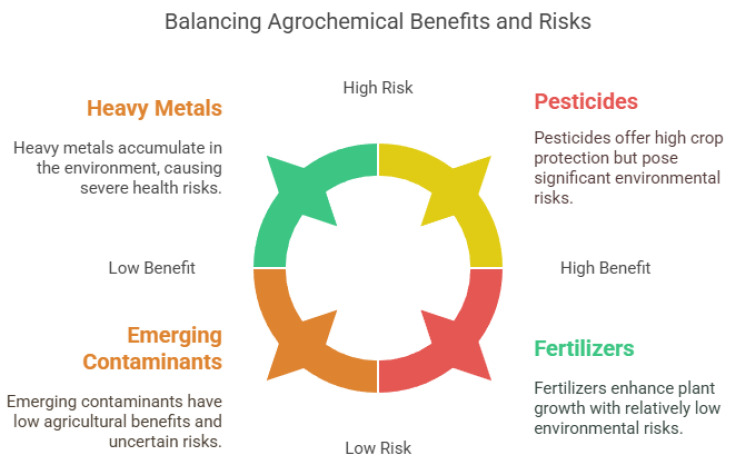
Benefits and risks of agrochemicals, including heavy metals, pesticides, emerging contaminants, and fertilizers.

Persistent Organic Pollutants (POPs), such as pesticides, PCBs, and PAHs, pose serious health and environmental risks due to their stability, bioaccumulation, and toxicity [70]. Originating from industrial waste, pesticides, and fossil fuel combustion, they persist in air, water, and soil, leading to widespread contamination [71]. POP exposure is linked to cancer, endocrine disruption, neurotoxicity, immune suppression, and cardiovascular and developmental disorders via oxidative stress and DNA damage [72,73]. These pollutants also accumulate in ecosystems, disrupting biodiversity and contaminating food webs. Agriculture heavily depends on various chemical substances to boost crop yields and protect plants from pests and diseases. These common substances, known as agrochemicals, include pesticides, herbicides, fungicides, and fertilizers (Figure 2) [3,74]. Although they are essential in modern farming, their widespread use poses significant risks to human health and the environment [75]. Fertilizers are nutrient-rich compounds that promote plant growth [76]. Overusing nitrogen-based fertilizers can lead to water contamination and eutrophication [77]. Organochlorine pesticides degrade soil health, PCBs are linked to neurological and reproductive issues [78], and PAHs pose ecotoxicological risks in aquatic environments [79,80]. Bioremediation methods like microbial and enzymatic degradation show promise but are limited by site-specific conditions [81]. While effective in crop protection, pesticides can remain in the environment, contaminating soil, water, and air [82]. Herbicides are used to control unwanted plants [83]. Fungicides prevent or eliminate fungal infections [84]. Nitrogen- and phosphorus-based fertilizers are commonly used to enhance plant growth, but excessive use can cause nutrient runoff into water bodies, leading to eutrophication and harmful algal blooms [85]. The accumulation of toxic compounds in soil can reduce soil fertility, disrupt microbial communities, and lead to biodiversity loss [77]. Agrochemicals can leach into groundwater or run off into surface water, contaminating drinking water sources and aquatic ecosystems [86]. Exposure to toxic compounds in agriculture can occur through inhalation, ingestion, or skin contact, leading to health effects such as endocrine disruption, carcinogenicity, reproductive disorders, and neurological issues [87].

Another common class of POPs in environments, particularly in food, is mycotoxins, which are extremely harmful substances created by different fungi that can accumulate in crops during growing and after harvesting. A specific type of mycotoxin, aflatoxins (AFs), is produced by specific *Aspergillus* sp., including *A. flavus*, *A. nomius*, and *A. parasiticus* [88,89]. Environmental factors, including food composition, high temperatures, extended periods of drought, prolonged storage, and poor storage conditions, significantly influence the proliferation of these fungi and the subsequent production of AFs [90,91]. Among the twenty aflatoxins (AFs), four are particularly notable: AFG2 (aflatoxin G2), AFG1 (aflatoxin G1), AFB2 (aflatoxin B2), and AFB1 (aflatoxin B1) [92]. The IARC identifies AFB1 as the most hazardous variant, known for its hepatotoxic, carcinogenic, and mutagenic properties [93,94]. Extended exposure to this toxic mycotoxin can accumulate in the body, possibly resulting in chronic health issues, especially liver cancer [95]. The European Union (EU) has set aflatoxin (AF) limits at 2 μg kg^−1^ for AFB1, the most toxic mycotoxin, and 4 μg kg^−1^ for the combined total of all classes of aflatoxin in cereals and related products [96].

Biogenic amines (BAs) are low-molecular-weight bioactive nitrogen compounds present in microorganisms, animals, and plants. They have various chemical structures and are classified into three primary categories: aliphatic polyamines, aliphatic diamines, and aromatic amines [97]. BAs are formed in food mainly by three factors: (1) lactic acid bacteria with decarboxylation activity; (2) the quality and characteristics of the raw material, including ionic strength, composition, and pH; and (3) processing and storage conditions, such as refrigeration, curing, or fermentation [98]. Many studies indicate that eating seafood with more than 500 mg kg^−1^ of histamine can lead to food poisoning [99]. The FDA (Food and Drug Administration) establishes 100 mg kg^−1^ for tyramine and histamine at 50 mg kg^−1^ in fish as admissible limits [100].

Polycyclic aromatic hydrocarbons (PAHs) are recognized as chemical pollutants due to their lipophilic and hydrophobic properties; these compounds primarily consist of aromatic rings containing carbon and hydrogen [101]. They typically arise or accumulate in food during thermal processing from the pyrolysis of organic materials or incomplete combustion [102]. Notably, benzo(a)pyrene (BaP), a specific type of PAH, has been linked to harmful effects on the human body. Prolonged exposure to PAHs from breathing, smoking, and eating contaminated food and water can accumulate in different organs, causing DNA damage, tumor formation, and a higher risk of lung, colon, and breast cancers. [103]. The EFSA reported a concentration higher than 1600 mg kg^−1^ for PAHs as a potential acute toxicity risk [104].

### 4.3. Emerging Contaminants

Emerging contaminants (ECs)—including pharmaceuticals, microplastics, and personal care products—are increasingly found in ecosystems due to wastewater, industrial discharge, and runoff [105]. Pharmaceuticals (e.g., trimethoprim and diclofenac) contribute to antimicrobial resistance and endocrine disruption [106,107]. Microplastics act as pollutant carriers and enter food chains [108,109,110]. Personal care product residues, such as phthalates and surfactants, disrupt hormonal functions and harm aquatic life [107,111]. These contaminants are not traditionally regulated but have recently been recognized as potential environmental pollutants [112]. In the case of food safety, contaminants are divided into intentional additives, which enhance food quality, and incidental contaminants, which arise unintentionally during food processing [113]. Eight compounds with significant toxic effects on human health in trace amounts include aflatoxin, HMF (hydroxymethylfurfural), BAs (biogenic amines), AA (acrylamide), furfural, PAHs (polycyclic aromatic hydrocarbons), BPA (bisphenol A), NAs (nitrosamines) [114].

The Maillard and caramelization reactions involve a series of chain reactions that produce appealing flavors and aromas in foods during various high-temperature cooking processes [115]. Hydroxymethylfurfural (HMF) and furfural (F) are cyclic aldehydes resulting from the breakdown of hexoses in caramelization and the Maillard reaction, acting as toxic byproducts [116]. HMF levels serve as a key quality indicator for assessing the intensity of heat treatment and the storage duration in a variety of food products [117]. Few studies have pinpointed distinct clinical symptoms related to F and HMF consumption. Nonetheless, specific research suggests that increased HMF concentrations may cause cytotoxic effects, irritating the skin, mucous membranes, and eyes [118]. The Codex Alimentarius states that the ADI value for furfural is 0.5 mg kg^−1^. Additionally, a level of HMF exceeding 40 mg kg^−1^ is considered toxic to humans [119].

Acrylamide (AA) is a polar, low-volatile, and hydrophilic unsaturated amide [120]. AA is an unavoidable toxic byproduct of the Maillard reaction, resulting from the formation of desirable aromas, colors, and flavors. Its production occurs through the chemical interaction of reducing sugars, such as glucose and fructose, with asparagine, a free amino acid, during heating processes like roasting, frying, and baking at temperatures exceeding 120 °C [121,122,123]. Carbohydrate-rich items, particularly plant-based foods, are significant contributors to acrylamide formation. AA is categorized as a neurotoxic, genotoxic, mutagenic, and carcinogenic contaminant. It can gradually affect different body parts, particularly the cardiovascular and renal systems, thereby impacting human health [124,125]. An intake per day of AA above 40 μg kg^−1^ is being reported for neurotoxic effects and 2.6 μg kg^−1^ for carcinogenic effects [126].

Bisphenol A (BPA) serves as the monomer for polycarbonate (PC), a material commonly found in the plastic industry, especially in food and beverage packaging [127]. The increasing number of ready-to-eat meals and canned goods on a global level signifies a higher demand for BPA in these packaging. BPA leaches into food and subsequently enters the human body. Typically, the primary pathway for human exposure to BPA correlates with the consumption of canned foods [128,129]. Factors such as the storage duration and heating or freezing processes influence the amount of BPA that migrates from bottles or cans into the food [130]. BPA is known to have toxic effects on the human genome, reproductive capabilities, and the neurological, immune, and cardiovascular systems, also acting as a potential carcinogen [131,132]. The European Commission (EC) has proposed a TDI for BPA at 50 μg kg^−1^, along with food-specific migration limits set at 0.6 mg kg^−1^ [133]. N-nitrosamine is a harmful compound generated from nitrates and nitrites, which are commonly added to certain foods, especially meat products. These substances not only enhance the quality of meat by acting as antibacterial preservatives and colouing agents but also contribute to its desirable flavor [134,135]. The primary way that N-nitrosamines (NAs) are formed is through the reaction of nitrates and nitrites with secondary amines found in food. Various factors affect the development of NAs in food, including acidity, cooking techniques, the duration, the alkalinity of secondary amines, nitrite levels, and the presence of catalysts or inhibitors [136,137]. Epidemiological studies classify NAs as food toxins that can lead to uncontrolled cell growth, consequently resulting in tumors in various organs, including the bladder, lungs, liver, pancreas, and esophagus [138,139]. In the USA, there is a daily maximum limit of 10 μg kg^−1^ per body weight in retail food products [140].

## 5. Applications of CND-Based Sensors for Detecting Toxic Compounds

### 5.1. Applications of CND-Based Sensors for Detecting Heavy Metals

Carbon nanodots (CNDs) have significantly attracted their usage in the food industry, which is mainly due to their fluorescence under UV light. This fluorescence emitted either increases or quenches with specific types of ions/molecule or substances [141,142]. Within certain limits, the quencher’s concentration is equal to the fluorescence intensity emitted [143,144]. CNDs marks their potential usage as efficient and sensitive probes for the detection of different types of analytes in food systems [145]. Table 2 summaries the different CND synthesis methods, including hydrothermal and microwave assisted with various precursors, such as gallic acid, citric acid, and o-phenylenediamine. CNDs are typically characterized using spectroscopic techniques such as FT-IR, UV-Vis, and FS; microscopic techniques (TEM, HR-TEM, SEM, and AFM); and surface analysis techniques (XPS, Zeta potential, DLS, XRD, and EDX), ensuring detailed evaluation of their morphology, structure, and surface functionality. For example, CNDs have been effectively used to detect heavy metal ions such as As^5+^, Fe^2+^, Hg^2+^, and Fe^3+^, with limits of detection (LOD) reported at 31.50 μM, 122.4 μM, 96.40 μM, and 161.9 μM, respectively (Table 3 [146]). Their strong linearity (*R*^2^ > 0.99) further supports their potential in food safety monitoring. Sensors based on CNDs have shown significant potential in detecting various toxic substances, including heavy metals, in agricultural environments (Table 4). CNDs can detect heavy metals through mechanisms such as fluorescence quenching and electrochemical sensing (Figure 2; [147]). When CNDs interact with heavy metal ions, changes in their photoluminescence properties are observed, which can be used to determine the presence and concentration of metals like lead, cadmium, and mercury [148,149]. This makes CND-based sensors highly effective for detecting heavy metal contamination in soil and water, providing a fast, sensitive, and cost-effective method for monitoring agricultural environments.

### 5.2. Applications of CND-Based Sensors for Detecting Organic Pollutants

In addition to heavy metals, CNDs have shown excellent performance in detecting organic pollutants in food systems. CNDs can be used as active ingredients in smart packaging materials to improve packaging quality and detect microbial deterioration [150]. Traditional detection methods like spectroscopy and chromatography [151,152] offer high accuracy but require costly equipment, complex sample preparation, and skilled personnel. In contrast, CND-based fluorescent probes provide a cheap, simple, rapid, and eco-friendly alternative. CNDs have been successfully applied for the detection of food additives and toxins such as melamine and acrylamide, achieving nanomolar sensitivity with low detection limits and strong linear relationships (*R*^2^ > 0.99). Their biocompatibility, low toxicity, and flexibility in fabrication methods enhance their suitability for widespread applications in detecting various organic food pollutants [153].

Furthermore, these sensors can identify even trace amounts of pesticides in soil and water, offering a quick and sensitive method for monitoring pesticide residues [154]. The fluorescence properties of CNDs allow them to be functionalized selectively to bind with organic pollutants, facilitating efficient detection and improving environmental monitoring practices. This is particularly important in agriculture, where the safe and controlled use of pesticides is crucial to prevent contamination of food and the environment.

### 5.3. Applications of CND-Based Sensors for Detecting Emerging Contaminants

Emerging contaminants like toxic polymer compounds, endocrine-disrupting chemicals, and allergens have also become a focus for CND-based sensor applications. Various fabrication methods—such as hydrothermal, microwave-assisted, solvothermal, gamma irradiation, and sol–gel techniques (Table 2, [50,155])—allow for tuning the properties of CNDs, impacting their sensitivity and selectivity [156]. CNDs have demonstrated strong potential in detecting contaminants like Aflatoxin M1, with LODs reported between 0.02 and 0.07 μg L^−1^ [157,158]. Moreover, CNDs offer an eco-friendly and low-cost option compared to conventional fluorescent probes made from expensive and toxic materials. Despite these advances, challenges remain regarding the cytotoxicity, bioaccumulation, and regulatory approval of CNDs. Future research must focus on sustainable large-scale production, ensuring stability, specificity, and safety for broader food industry applications. CND-based sensors can be used to monitor the presence and concentration of emerging contaminants in agricultural settings, ensuring safer practices and better management of environmental pollution [159]. CNDs have emerged as powerful tools in detecting antibiotic resistance. Their versatility, cost-effectiveness, and adaptability make them promising candidates for advancing diagnostic and therapeutic strategies in antibiotic resistance management which is summarized in Table 5.

**Table 2:** Comprehensive summary of different CND synthesis methods with various precursors and their characterization techniques.

Method of Synthesis	Precursors Used	Characterization Techniques Used	References
Hydrothermal	Gallic acid and DMF	FT-IR, DLS, HR-TEM, XRD, XPS, and FS	[160]
	Tea bag waste	UV–Vis, PSA, Zeta potential, HR-TEM, AFM, FT-IR, DSC, and FS	[161]
	o-phenylenediamine, dipicolinic acid	HR-TEM, DLS, FT-IR, XPS, and FS	[162]
	o-phenylenediamine	FT-IR, DLS, TEM, XRD, SEM, and XPS	[163]
	*Salvadora persica* powder and m-phenylenediamine	TEM, Zeta potential, UV-Vis, FT-IR, XPS, TRPL, and FS	[164]
	Citric acid and polyethyleneimine	HR-TEM, EDX, FT-IR, UV-Vis, and FS	[158]
	Citric acid and ethylenediamine	TEM, XPS, FT-IR, UV-Vis, and FS	[157]
	Ethylene glycol and sodium hydroxide	UV-Vis, TEM, FT-IR, and FS	[165]
	Citric acid, arginine, and ethane diamine	TEM, FT-IR, Zeta potential, XPS, UV-Vis, and FS	[166]
Microwave assisted	Citric acid, urea, and trisodium citrate	FT-IR, UV-Vis, XRD, FS, HR-TEM, and RS	[167]
	Cerium nitrate, dopamine hydrochloride, and citric acid	FT-IR, XPS, XRD, TEM, RS, UV-Vis, EPR, and Zeta potential	[168]
Solvothermal	Neutral red, sulfuric acid and glutathione	HR-TEM, SEM, FT-IR, XPS, PXRD, and DSC	[40]
Gamma irradiation	Sucrose and ammonia	Zeta potential, FT-IR, UV-Vis, XRD, XPS, TEM, and FS	[146]
Pyrolysis, sol–gel, and electrodeposition	Titanium oxide and citric acid	FT-IR, EM, Zeta analysis, UV–Vis, and XRD	[169]
Oil bath	Sucrose and urea	UV-Vis, FS, TEM, FT-IR, XRD, and XPS	[170]

**Table 3:** Application of CNDs for the detection of toxic compounds in food systems.

	Analyte Detected	LOD	Calibration Range	References
Metal ions	As^5+^, Fe^2+^, Hg^2+^, and Fe^3+^	As^5+^—31.50 μM, Fe^2+^—122.4 μM, Hg^2+^—96.40 μM, and Fe^3+^—161.9 μM	As^5+^—0.09–0.19 mM (*R*^2^ = 0.9969), Fe^2+^—0.01–0.8 mM (*R*^2^ = 0.9966), Hg^2+^—0.04–0.9 mM (*R*^2^ = 0.9962), and Fe^3+^—0.01–0.9 mM (*R*^2^ = 0.9967)	[146]
	Pb^2+^	0.715 μM	30–130 μM (*R*^2^ = 0.9902)	[160]
	Fe^3+^ and Ag^+^	Fe^3+^—0.250 μM; Ag^1+^—0.140 μM	Fe^3+^—1–100 μM (*R*^2^ = 0.9952); Ag^1+^—1–200 μM (*R*^2^ = 0.9985)	[167]
	Hg^2+^	0.147 µg L^−1^	0.625–90 µg L^−1^ (*R*^2^ = 0.9960)	[168]
Polymer	Melamine	30 nM	0–20 μM (*R*^2^ = 0.9940)	[165]
	Melamine	0.67 μM	2.0 to 290 μM (*R*^2^ = 0.9981)	[166]
	Acrylamide	0.354 μg L^−1^	0.5–10 μg L^−1^ (*R*^2^ = 0.9991)	[161]
	Acrylamide	0.670 nM	10–200 nM (*R*^2^ = 0.9876)	[169]
	L-asparagine	0.31 μM	1.0–50.0 μM (*R*^2^ = 0.9984)	[170]
Food Additives	Erythrosine	1.210 nM	4–20 µM (*R*^2^ = 0.9970)	[164]
	Malachite green	1.200 nM	0.014–300 µM (*R*^2^ = 0.9964)	[40]
Mycotoxin	Aflatoxin M1	0.07 μg L^−1^	0.2–0.8 μg L^−1^ (*R*^2^ = 0.9552)	[158]
Gamma irradiation	Aflatoxin M1	0.0186 μg kg^−1^	0.003–0.81 μg kg^−1^ (*R*^2^ = 0.9940)	[157]
Insecticide	Imidacloprid	1.870 μg kg^−1^	0.037–0.2 mg kg^−1^ (*R*^2^ = 0.9700)	[163]
Allergen	Histamine	6.96 µM	25–1000 µM (*R*^2^ = 0.9978)	[162]

**Table 4:** Application of carbon nanodots (CNDs) in detecting and monitoring pesticide residues.

Source of Carbon Dots	Studied Pesticides	References
Green-fluorescent C-dots from vegetables/fruits	Pesticide parathion methyl can be detected through the reliable and sensitive technique in real food samples	[171]
Porphyrinic Zr metal–organic framework (PCN-224@CDs)	Organo-phosphorus pesticides via carbon dots supported Zr-based metal organic framework	[172]
Producing carbon quantum dots from tea residue	Using Al_2_(SO_4_)_3_/CQDs composite in photocatalytic degradation of pesticide Fipronil (C_12_H_4_C_l2_F_6_N_4_OS)	[173]
Using carbon dots from *Boerhavia diffusa* leaves	Nanocomposite of CDs/cobalt ferrite and boehmite for photo-degradation via sensing of pesticide methyl parathion	[174]
Producing carbon dots using gallic acid	Detecting the “organophosphate pesticide” chlorpyrifos in wastewater as fluorescent probe	[175]
Producing carbon dots using *Grewia asiatica* fruit via microwave	Detecting the organo-phosphorus pesticide of quinalphos by forming red emissive carbon dots in vegetable, water, and soil samples	[176]
Bio-producing CeO_2_@C-dots from agrowaste (chestnut peels)	Active catalyst for degradation of Rhodamine B dye and detecting 4-Nitrophenol in aqueous solutions	[177]
Nitrogen-doped carbon dots using hydrothermal method	Detecting imidacloprid as an effective neonicotinoid insecticide in real foods	[178]
Boron-nitrogen doped carbon dots (BN-C-dots) using hydrothermal protocol	Rapid detection of insecticide acephate using BN-C-dots in vegetables and water	[179]
Producing TiO_2_/ZnO-CQDs from spent coffee using hydrothermal method	Detecting carbaryl (C_12_H_11_NO_2_) in carbamate pesticides via photocatalytic degradation in water for environmental remediation approach	[180]
Producing graphene carbon dots in corn stalk pith as green sorbent	Using the studied green sorbent in detecting triazole fungicide residues in different types of rice samples	[181]
Nitrogen- and phosphorus-doped carbon quantum dots (NP-CQDs) via hydrothermal method	Using fluorescent NP-CQDs in detecting pesticides of chlorpyrifos in miscellaneous beans and their residues in different kinds of foods	[41]
Nitrogen-doped carbon dots (N-CDs)	Using N-CD-based fluorescent sensor for detecting glyphosate in organo-phosphorus pesticides	[182]

**Table 5:** Application of carbon nanodots (CNDs) in detecting or combating antibiotic resistance.

Source of Carbon Dots	Studied Antibiotics	Ref.
Producing N- and S-doped blue-fluorescent carbon dots via a one-step solvothermal protocol	Detecting chloramphenicol in environmental and food safety contexts as portable fluorescence-based sensor for several analytical purposes	[183]
Producing N-doped C-quantum dots via microwave-assisted hydrothermal protocol	Detecting the antibiotic meropenem using a sensor of N-CQDs-AuNPs in pharmaceuticals and plasma	[184]
Producing N-doped carbon quantum dots using hydrothermal protocol	N-CQDs are more effective than antibiotic levofloxacin in treating bacterial keratitis caused by multidrug-resistant *Staphylococcus aureus*, combating its resistance	[185]
Producing Curcumin-derived C-dots via hydrothermal protocol	Cur-CDs exhibit significant antibacterial effects against strains such as *Escherichia coli*, and *Staphylococcus aureus* comparing with antibiotic chloramphenicol	[186]
Producing fluorescent C-quantum dots from disposable water bottles	Using Polyethylene Terephthalate plastic (PET) fluorescent C-quantum dots for removing antibiotic ciprofloxacin	[187]
Producing N and S co-doped C-quantum dots using hydrothermal protocol	On-site detection of antibiotics (i.e., moxifloxacin, gatifloxacin, and ofloxacin) in milk using N, S co-doped CQNs in combination with a smartphone	[188]
Producing N-doped C-quantum dots from the peels of *Citrus limetta*	Detecting β-Lactam antibiotics (ampicillin) in milk and water depending on the N-CQDs by forming a greenish-blue fluorescent color	[189]
Producing highly water-soluble Curcumin carbon dots using the hydrothermal protocol	Studied Cur-CDs exhibited a higher antimicrobial efficacy against *Escherichia coli* and *Staphylococcus aureus* and combating drug-resistant bacterial infections	[190]
Producing N-doped green-fluorescent C-dots using the hydrothermal protocol	A cost-effective, reliable approach, using high-fluorescence CDs for detecting the antibiotic chlortetracycline in real samples	[191]
Producing stable red-fluorescent nitrogen-doped carbon dots via solvothermal method	Studying N-CDs as a fluorescent probe to detect ceftazidime antibiotic in real samples	[192]
Producing CDs via hydrothermal method using different quaternary ammonium salts	Detecting tetracycline antibiotics in real milk using CDs as a cost-effective approach for providing insights into food safety testing methodologies	[193]
Producing magnetic molecular nanomaterials coupled with CDs via hydrothermal method	Detecting doxycycline antibiotics using CDs in food matrices through fluorescence quenching mechanism and inner filter effect (IFE)	[194]

## 6. Sensitivity, Selectivity, and Performance of CND-Based Sensors

**Figure 3:**
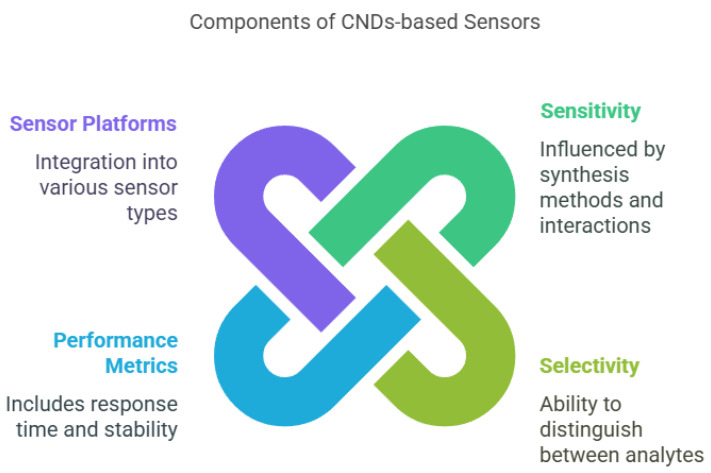
The main components of CND-based sensors.

The development of sensors based on carbon nanodots (CNDs) marks a significant leap in nanotechnology, providing high sensitivity, selectivity, and performance for detecting various analytes (Figure 3; [17,195]. These parameters are essential for applications in biosensing, environmental monitoring, and food safety [196]. Sensitivity, defined as the ratio of the change in sensor output to the change in input signal, reflects how responsive the sensor is to low concentrations of analytes [197]. High sensitivity in CND-based sensors can be attributed to their strong fluorescence, high surface area, and efficient interaction with target molecules [198]. Several factors influence the sensitivity, including synthesis methods (e.g., hydrothermal, solvothermal, microwave assisted, and electrochemical), which determine the size, shape, and surface properties of the CNDs [199]. Smaller, uniformly distributed CNDs often offer higher sensitivity due to a larger surface area-to-volume ratio [200]. Surface functionalization, such as with carboxyl, hydroxyl, or amine groups, enhances sensitivity by facilitating stronger interactions with analytes [201]. Environmental parameters, like the pH, temperature, and ionic strength, also affect the performance of the sensor and must be optimized for consistent detection [202]. The choice of detection mechanism—fluorescence quenching, electrochemical response, or colorimetric changes—also influences the sensitivity, with fluorescence-based detection being particularly popular due to its simplicity and real-time response [203].

Selectivity, which is the ability of a sensor to distinguish a specific analyte in the presence of other substances, is crucial in complex environments like biological fluids or food matrices [204]. It can be significantly enhanced through the surface modification of CNDs with recognition elements such as antibodies, enzymes, or molecularly imprinted polymers [205]. These modifications create specific binding sites that improve their interaction with target analytes. The synthesis method and environmental conditions (e.g., pH and ionic strength) also affect the selectivity by altering the binding affinity and specificity [206]. Furthermore, the detection mechanism can influence the selectivity: for example, electrochemical sensors may be more selective for redox-active compounds, while fluorescence sensors may be designed to respond only to specific fluorophore-target interactions [207].

The performance of CND-based sensors also depends on other factors, such as the response time, stability, reproducibility, and detection limit [208]. A fast response time is critical for real-time monitoring, while long-term stability ensures consistent performance under varying environmental conditions [209]. Reproducibility, or the sensor’s ability to deliver consistent results across multiple measurements or batches, is essential for reliable use [210]. The detection limit, which indicates the lowest concentration of analyte the sensor can detect, is particularly important for applications requiring high precision, such as medical diagnostics or trace contaminant detection in food and water [211,212].

**Figure 4:**
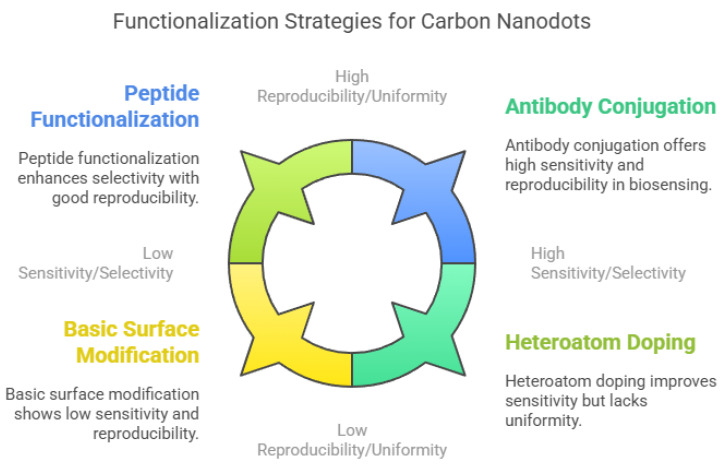
Functionalization strategies of carbon nanodots (CNDs).

To enhance sensitivity and selectivity, researchers have developed various functionalization strategies (Figure 4). Bio-functionalization with antibodies, aptamers, or peptides is a promising approach, as these biomolecules provide high binding affinity and specificity toward target analytes. For instance, CNDs conjugated with horseradish peroxidase (HRP)-linked antibodies showed enhanced sensitivity for detecting carcinoembryonic antigen (CEA) [213,214,215]. Selective peptides have also improved the detection of specific pollutants and biomolecules [216]. Doping CNDs with heteroatoms such as nitrogen or sulfur can modulate their electronic structure and enhance their photoluminescence, thereby increasing sensor sensitivity and stability [217]. Chemical modification techniques like click chemistry enable site-specific functionalization for targeted applications [218]. Both covalent and non-covalent approaches are used to introduce functional groups or recognition elements to the CND surface. However, challenges remain in achieving uniform functionalization and maintaining reproducibility across batches [201]. Developing standardized protocols for functionalization will be crucial for advancing CND-based sensors into scalable, real-world applications in biomedical diagnostics, environmental monitoring, and food safety [156].

## 7. Challenges and Future Perspectives

**Figure 5:**
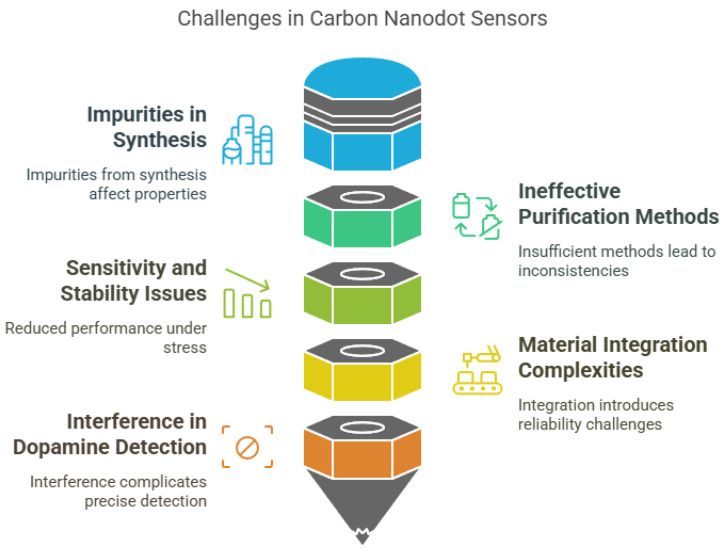
Challenges in carbon nanodot-based sensors.

Despite the promising potential of carbon nanodot (CND)-based sensors, several limitations continue to hinder their widespread application in real-world scenarios. These challenges span across material synthesis, performance stability, functional complexity, and scalability, all of which impact the sensors’ consistency, cost effectiveness, and reliability in diverse environments (Figure 5). One of the most persistent issues lies in the synthesis and purification of CNDs. Like other carbon-based nanomaterials such as graphene quantum dots (GQDs) and carbon nanotubes (CNTs), the production of CNDs often results in impure products containing small molecules and aggregates, which significantly alter their physicochemical properties and interfere with sensor performance [219]. The purification methods currently available are insufficient to eliminate these impurities entirely, leading to inconsistencies in reproducibility and sensitivity [219].

Performance-related limitations are also a concern. CND-based sensors may exhibit reduced sensitivity and stability, particularly in flexible or wearable applications, where they are subject to mechanical stress [220]. Although integrating CNDs with other materials, such as polyaniline, can enhance overall sensor capabilities, it often introduces structural and functional complexities that complicate sensor fabrication and may compromise stability [220]. Furthermore, certain applications—such as dopamine detection—face challenges due to the potential for interference from similar biomolecules, making precise quantification difficult [221]. In addition, the intricate surface functionalization required for improving selectivity and target specificity increases production costs and reduces practicality for commercial deployment [222].

**Figure 6:**
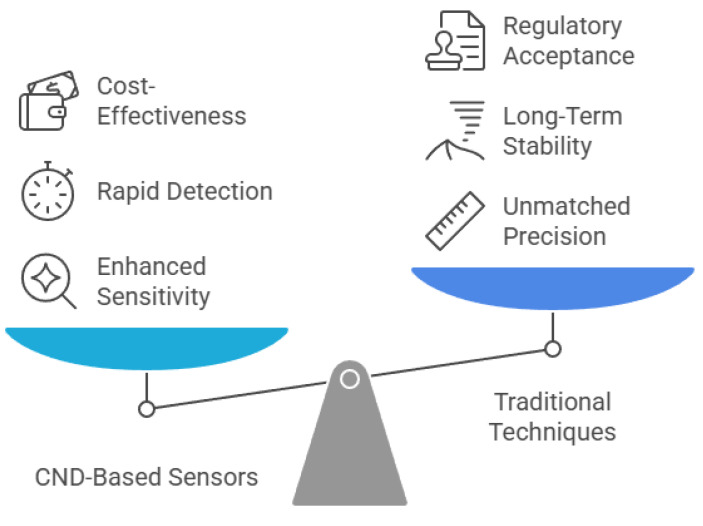
Balancing innovation and tradition in carbon nanodot-based sensors.

Comparatively, CND-based sensors share both advantages and limitations with other carbon nanomaterial sensors and traditional analytical techniques (Figure 6). They offer rapid detection, high sensitivity, and low detection limits, making them attractive for real-time environmental monitoring. For instance, compared to techniques like inductively coupled plasma mass spectrometry (ICP-MS) and high-performance liquid chromatography (HPLC), CND-based sensors can deliver near-instantaneous results with minimal sample preparation [223]. Their lower production and operational costs also make them suitable for deployment in resource-limited settings [110]. However, selectivity remains a common limitation among carbon nanomaterial-based sensors, often leading to cross-reactivity and false positives when detecting analytes in complex matrices [224]. Additionally, the fabrication of stable and reproducible CND-based sensor platforms typically requires advanced nanofabrication techniques, which limits their scalability [225]. Long-term stability is another issue, as environmental factors such as humidity, temperature changes, and chemical interference can degrade sensor performance over time [226].

Looking ahead, future research should focus on overcoming these challenges through the development of multifunctional sensors, advanced hybrid materials, and novel synthesis and purification techniques. Incorporating artificial intelligence (AI)-assisted data processing may also improve signal interpretation and enhance sensor accuracy in complex environments. Moreover, the integration of CND-based sensors into smart, portable devices opens new avenues for real-time diagnostics and environmental surveillance. While traditional methods like ICP-MS and HPLC remain the gold standard for precision and regulatory approval, the ongoing evolution of carbon nanomaterials holds significant promise for expanding the practical utility and commercial scalability of CND-based sensing technologies.

## 8. Conclusions

Carbon nanodot-based sensors represent a significant advancement in the field of analytical detection technologies, offering unique advantages in sensitivity, selectivity, and environmental compatibility. Their successful application in identifying toxic compounds across food, agricultural, and environmental matrices demonstrates their versatility and efficacy. With customizable surface functionalities, tunable optical properties, and compatibility with diverse detection platforms, CNDs provide a foundation for next-generation sensing devices. However, challenges such as inconsistent synthesis, limited scalability, and long-term stability must be addressed to fully harness their potential. Future innovations should focus on refining green synthesis methods, improving functionalization strategies, and developing integrated portable sensor systems for real-time, on-site monitoring. The continued convergence of nanotechnology, materials science, and environmental engineering will pave the way for deploying CND-based sensors as essential tools in ensuring public health, food security, and ecological resilience.

## References

[1] Chatterjee R. (2009). Economic Damages from Nutrient Pollution Create a “Toxic Debt”. Environ. Sci. Technol..

[2] Macêdo R.L., Haubrock P.J., Klippel G., Fernandez R.D., Leroy B., Angulo E., Carneiro L., Musseau C.L., Rocha O., Cuthbert R.N. (2024). The Economic Costs of Invasive Aquatic Plants: A Global Perspective on Ecology and Management Gaps. Sci. Total Environ..

[3] Alengebawy A., Abdelkhalek S.T., Qureshi S.R., Wang M.-Q. (2021). Heavy Metals and Pesticides Toxicity in Agricultural Soil and Plants: Ecological Risks and Human Health Implications. Toxics.

[4] Thompson L.A., Darwish W.S. (2019). Environmental Chemical Contaminants in Food: Review of a Global Problem. J. Toxicol..

[5] Carvalho F.P. (2017). Pesticides, Environment, and Food Safety. Food Energy Secur..

[6] Jan A.T., Azam M., Siddiqui K., Ali A., Choi I., Haq Q.M.R. (2015). Heavy Metals and Human Health: Mechanistic Insight into Toxicity and Counter Defense System of Antioxidants. Int. J. Mol. Sci..

[7] Molina J., Cases F., Moretto L.M. (2016). Graphene-Based Materials for the Electrochemical Determination of Hazardous Ions. Anal. Chim. Acta.

[8] Álvarez-Ruiz R., Picó Y. (2020). Analysis of Emerging and Related Pollutants in Aquatic Biota. Trends Environ. Anal. Chem..

[9] Ullah N., Mansha M., Khan I., Qurashi A. (2018). Nanomaterial-Based Optical Chemical Sensors for the Detection of Heavy Metals in Water: Recent Advances and Challenges. TrAC Trends Anal. Chem..

[10] Sharma S., Bhattacharya A. (2017). Drinking Water Contamination and Treatment Techniques. Appl. Water Sci..

[11] Sillanpää M., Ncibi M.C., Matilainen A., Vepsäläinen M. (2018). Removal of Natural Organic Matter in Drinking Water Treatment by Coagulation: A Comprehensive Review. Chemosphere.

[12] Qu X., Alvarez P.J.J., Li Q. (2013). Applications of Nanotechnology in Water and Wastewater Treatment. Water Res..

[13] Wang L., Kim J., Cui T. (2018). Self-Assembled Graphene and Copper Nanoparticles Composite Sensor for Nitrate Determination. Microsyst. Technol..

[14] Sankaranarayanan A., Amaresan N., Sharma A., Khalifa A.Y.Z. (2021). Mycotoxins Associated Food Safety Concerns of Agricultural Crops, Prevention and Control. Fungi Bio-Prospects in Sustainable Agriculture, Environment and Nano-Technology.

[15] Molla A., Youk J.H. (2023). Recent Progress on Electroanalytical Sensing of Small Molecules and Biomolecules Using Carbon Dots: A Review. J. Ind. Eng. Chem..

[16] Li H., Kang Z., Liu Y., Lee S.-T. (2012). Carbon Nanodots: Synthesis, Properties and Applications. J. Mater. Chem..

[17] Dhamodharan D., Byun H.-S., Varsha Shree M., Veeman D., Natrayan L., Stalin B. (2022). Carbon Nanodots: Synthesis, Mechanisms for Bio-Electrical Applications. J. Ind. Eng. Chem..

[18] Etefa H.F., Tessema A.A., Dejene F.B. (2024). Carbon Dots for Future Prospects: Synthesis, Characterizations and Recent Applications: A Review (2019–2023). C.

[19] Mansuriya B.D., Altintas Z. (2021). Carbon Dots: Classification, Properties, Synthesis, Characterization, and Applications in Health Care—An Updated Review (2018–2021). Nanomaterials.

[20] Liu J., Li R., Yang B. (2020). Carbon Dots: A New Type of Carbon-Based Nanomaterial with Wide Applications. ACS Cent. Sci..

[21] Ge G., Li L., Wang D., Chen M., Zeng Z., Xiong W., Wu X., Guo C. (2021). Carbon Dots: Synthesis, Properties and Biomedical Applications. J. Mater. Chem. B.

[22] Prokisch J., Törős G., Nguyen D.H.H., Neji C., Ferroudj A., Sári D., Muthu A., Brevik E.C., El-Ramady H. (2024). Nano-Food Farming: Toward Sustainable Applications of Proteins, Mushrooms, Nano-Nutrients, and Nanofibers. Agronomy.

[23] Bai J., Cheng Y., He F., Liu Q., Qiang S., Zhang L., Yang J., Wang C., Xu Y., Zhang W. (2022). Environment-Sensitive Carbon Dots Derived from Naphthalenediol for Solvent Polarity Indicator and Anti-Counterfeiting. ChemistrySelect.

[24] Lim S.Y., Shen W., Gao Z. (2015). Carbon Quantum Dots and Their Applications. Chem. Soc. Rev..

[25] Keçili R., Hussain C.G., Hussain C.M. (2023). Fluorescent Nanosensors Based on Green Carbon Dots (CDs) and Molecularly Imprinted Polymers (MIPs) for Environmental Pollutants: Emerging Trends and Future Prospects. Trends Environ. Anal. Chem..

[26] Das R., Shahnavaz Z., Ali M.E., Islam M.M., Abd Hamid S.B. (2016). Can We Optimize Arc Discharge and Laser Ablation for Well-Controlled Carbon Nanotube Synthesis?. Nanoscale Res. Lett..

[27] Kim M., Osone S., Kim T., Higashi H., Seto T. (2017). Synthesis of Nanoparticles by Laser Ablation: A Review. KONA.

[28] Shen S., Fu J., Wang H. (2019). A Facile, Effective Synthesis of Excellent Fluorescent Carbon Dots with Optical Properties. ChemistrySelect.

[29] Pawelski D., Plonska-Brzezinska M.E. (2023). Microwave-Assisted Synthesis as a Promising Tool for the Preparation of Materials Containing Defective Carbon Nanostructures: Implications on Properties and Applications. Materials.

[30] Stan C.S., Albu C., Coroaba A., Popa M., Sutiman D. (2015). One Step Synthesis of Fluorescent Carbon Dots through Pyrolysis of N-Hydroxysuccinimide. J. Mater. Chem. C.

[31] Barhoum A., Alhashemi Y., Ahmed Y.M., Rizk M.S., Bechelany M., Abdel-Haleem F.M. (2024). Innovations in Ion-Selective Optodes: A Comprehensive Exploration of Modern Designs and Nanomaterial Integration. Front. Bioeng. Biotechnol..

[32] Chen H., Luo K., Xie C., Zhou L. (2024). Nanotechnology of Carbon Dots with Their Hybrids for Biomedical Applications: A Review. Chem. Eng. J..

[33] Banger A., Gautam S., Jadoun S., Jangid N.K., Srivastava A., Pulidindi I.N., Dwivedi J., Srivastava M. (2023). Synthetic Methods and Applications of Carbon Nanodots. Catalysts.

[34] Kayani K.F., Ghafoor D., Mohammed S.J., Shatery O.B. (2024). Carbon Dots: Synthesis, Sensing Mechanisms, and Potential Applications as Promising Materials for Glucose Sensors. Nanoscale Adv..

[35] Roy S. (2024). Detection of Fluoride Ion by Carbon Dots-Based Fluorescent Probes. J. Mol. Struct..

[36] Shellaiah M., Sun K.W. (2023). Review on Carbon Dot-Based Fluorescent Detection of Biothiols. Biosensors.

[37] Abdel-Karim R. (2024). Nanotechnology-Enabled Biosensors: A Review of Fundamentals, Materials, Applications, Challenges, and Future Scope. Biomed. Mater. Devices.

[38] Zu F., Yan F., Bai Z., Xu J., Wang Y., Huang Y., Zhou X. (2017). The Quenching of the Fluorescence of Carbon Dots: A Review on Mechanisms and Applications. Microchim. Acta.

[39] Ma L., Liu J., Long X., Wu S. (2025). Green Synthesis of Fluorescent Carbon Dots from Waste Chicken Feathers for Chlortetracycline Sensing. J. Mol. Struct..

[40] Ali R., Elfadil H., Sirag N., Albalawi A.S., Albalawi A., Alharbi S., Al-anzi A., Alatawi S., Alhuaiti Y., Alsubaie F.T. (2025). A Novel Red Emissive Glutathione-Capped Carbon Dots Embedded within Molecularly-Imprinted Polymers for Adsorption and Fluorescent Sensing of Malachite Green in Food Samples. Microchem. J..

[41] Zhang L., Chen J., Zhang L., Yu R. (2025). Rapid Detection of Chlorpyrifos in Miscellaneous Beans Based on Nitrogen and Phosphorus Doped Carbon Quantum Dots Fluorescence Probe. J. Food Compos. Anal..

[42] Probst C.E., Zrazhevskiy P., Bagalkot V., Gao X. (2013). Quantum Dots as a Platform for Nanoparticle Drug Delivery Vehicle Design. Adv. Drug Deliv. Rev..

[43] Annamalai K., Ravichandran R., Annamalai A., Jeevarathinam A., Suresh R., Elumalai S. (2025). Synthesis of Blue-Sparkling N, S-Doped Carbon Dots for Effective Detection of Nitro Explosive and Fe^3+^ Ion and Anti-Counterfeiting Studies. Mater. Res. Bull..

[44] Duong P.V., Thi L.A., Hung P.Q., Toan L.D., Chuyen P.T., Hieu D.M., Minh P.H., Binh N.T., Cuong T.M., Hoa N.M. (2025). Probing Förster Resonance Energy Transfer in Carbon Quantum Dots for High-Sensitivity Aflatoxin B1 Detection. J. Fluoresc..

[45] Auria-Luna F., Foss F.W., Molina-Canteras J., Velazco-Cabral I., Marauri A., Larumbe A., Aparicio B., Vázquez J.L., Alberro N., Arrastia I. (2025). Supramolecular Chemistry in Solution and Solid–Gas Interfaces: Synthesis and Photophysical Properties of Monocolor and Bicolor Fluorescent Sensors for Barium Tagging in Neutrinoless Double Beta Decay. RSC Appl. Interfaces.

[46] Zhou Y., Huang X., Liu C., Zhang R., Gu X., Guan G., Jiang C., Zhang L., Du S., Liu B. (2016). Color-Multiplexing-Based Fluorescent Test Paper: Dosage-Sensitive Visualization of Arsenic(III) with Discernable Scale as Low as 5 Ppb. Anal. Chem..

[47] Chen S., Yu Y.-L., Wang J.-H. (2018). Inner Filter Effect-Based Fluorescent Sensing Systems: A Review. Anal. Chim. Acta.

[48] Ilie-Mihai R.-M., Gheorghe D.-C., Stefan-van Staden R.-I., Azad U.P., Chandra P. (2025). Carbon Nanodots in Nanobiomedicines and Electrochemical Sensing Devices. Handbook of Material Engineering in Nanobiomedicine and Diagnostics.

[49] Qian Y., Chen G., Ma C., Li L., Yang T., Zhu C., Gao H., Hu A., Guo X., Yang W. (2025). N-Doped Carbon Nanodots as Temperature Sensors and Fluorescent Probes for the Detection of Tinidazole in Milk. J. Fluoresc..

[50] Liu M.L., Chen B.B., Li C.M., Huang C.Z. (2019). Carbon Dots: Synthesis, Formation Mechanism, Fluorescence Origin and Sensing Applications. Green Chem..

[51] Mangaiyarkkarasi J. (2024). Harnessing Carbon Nanomaterials for Next-Generation Environmental Sensing. Environmental Applications of Carbon-Based Materials.

[52] Paramasivam G., Palem V.V., Meenakshy S., Suresh L.K., Gangopadhyay M., Antherjanam S., Sundramoorthy A.K. (2024). Advances on Carbon Nanomaterials and Their Applications in Medical Diagnosis and Drug Delivery. Colloids Surf. B Biointerfaces.

[53] Rabchinskii M.K., Ryzhkov S.A., Besedina N.A., Brzhezinskaya M., Malkov M.N., Stolyarova D.Y., Arutyunyan A.F., Struchkov N.S., Saveliev S.D., Diankin I.D. (2022). Guiding Graphene Derivatization for Covalent Immobilization of Aptamers. Carbon.

[54] Maurya N., Mishra S., Gupta M.K. (2024). Carbon Dots and Their Environmental Applications. Green Solutions for Degradation of Pollutants.

[55] Shaik R., Ghosh B., Barman H.C., Rout A., Padhy P.K. (2024). Green Nanotech: A Review of Carbon-Based Nanomaterials for Tackling Environmental Pollution Challenges. Nat. Environ. Pollut. Technol..

[56] Guo Y., Zhang Z., Guo X. (2024). Editorial: Recent Research Advances on Heavy Metals, Microplastics, Persistent Organic Pollutants, and Solid Waste in Aquatic and Terrestrial Ecosystems. Front. Environ. Sci..

[57] Luo Q. (2024). Impacts of Heavy Metal Pollution on Agricultural Production and Response Strategies. Sci. Technol. Eng. Chem. Environ. Prot..

[58] Sarma H.H., Rajkumar A., Baro A., Das B.C., Talukdar N. (2024). Impact of Heavy Metal Contamination on Soil and Crop Ecosystem with Advanced Techniques to Mitigate Them. JABB.

[59] Ullah H., Uddin J., Ijaz M., Haziq M., Muhsinah A.B., Ullah I., Jamal M. (2025). Metabolic Dynamics and Health Risk Assessment of Heavy Metal Accumulation in Urban–Rural Interface Vegetable Systems. Environ. Monit. Assess..

[60] Ma J., Hung H., Macdonald R.W. (2016). The Influence of Global Climate Change on the Environmental Fate of Persistent Organic Pollutants: A Review with Emphasis on the Northern Hemisphere and the Arctic as a Receptor. Glob. Planet. Chang..

[61] Balali-Mood M., Naseri K., Tahergorabi Z., Khazdair M.R., Sadeghi M. (2021). Toxic Mechanisms of Five Heavy Metals: Mercury, Lead, Chromium, Cadmium, and Arsenic. Front. Pharmacol..

[62] Malik L.A., Bashir A., Qureashi A., Pandith A.H. (2019). Detection and Removal of Heavy Metal Ions: A Review. Environ. Chem. Lett..

[63] Anderson A., Anbarasu A., Pasupuleti R.R., Manigandan S., Praveenkumar T.R., Aravind Kumar J. (2022). Treatment of Heavy Metals Containing Wastewater Using Biodegradable Adsorbents: A Review of Mechanism and Future Trends. Chemosphere.

[64] Saidon N.B., Szabó R., Budai P., Lehel J. (2024). Trophic Transfer and Biomagnification Potential of Environmental Contaminants (Heavy Metals) in Aquatic Ecosystems. Environ. Pollut..

[65] Nde S.C., Felicite O.M., Aruwajoye G.S., Palamuleni L.G. (2024). A Meta-Analysis and Experimental Survey of Heavy Metals Pollution in Agricultural Soils. J. Trace Elem. Miner..

[66] Xiong R., He X., Gao N., Li Q., Qiu Z., Hou Y., Shen W. (2024). Soil pH Amendment Alters the Abundance, Diversity, and Composition of Microbial Communities in Two Contrasting Agricultural Soils. Microbiol. Spectr..

[67] Moghimi Dehkordi M., Pournuroz Nodeh Z., Soleimani Dehkordi K., Salmanvandi H., Rasouli Khorjestan R., Ghaffarzadeh M. (2024). Soil, Air, and Water Pollution from Mining and Industrial Activities: Sources of Pollution, Environmental Impacts, and Prevention and Control Methods. Results Eng..

[68] Shirsat M.D., Hianik T. (2023). Electrochemical Detection of Heavy Metal Ions Based on Nanocomposite Materials. J. Compos. Sci..

[69] Yoo D., Park Y., Cheon B., Park M.-H. (2019). Carbon Dots as an Effective Fluorescent Sensing Platform for Metal Ion Detection. Nanoscale Res. Lett..

[70] Devi N.L., Saxena G., Bharagava R.N. (2020). Persistent Organic Pollutants (POPs): Environmental Risks, Toxicological Effects, and Bioremediation for Environmental Safety and Challenges for Future Research. Bioremediation of Industrial Waste for Environmental Safety: Volume I: Industrial Waste and Its Management.

[71] Akhtar A.B.T., Naseem S., Yasar A., Naseem Z., Prasad R. (2021). Persistent Organic Pollutants (POPs): Sources, Types, Impacts, and Their Remediation. Environmental Pollution and Remediation.

[72] Ahmed H., Sharif A., Bakht S., Javed F., Hassan W., Akash M.S.H., Rehman K. (2021). Persistent Organic Pollutants and Neurological Disorders: From Exposure to Preventive Interventions. Environmental Contaminants and Neurological Disorders.

[73] Said T.O., El Zokm G.M., Said T.O., El Zokm G.M. (2024). Toxicology and Ecological Risk with Emphasis on Scenario-Describing Mechanisms. Persistent Organic Pollutants in Aquatic Systems: Classification, Toxicity, Remediation and Future.

[74] Devi P.I., Manjula M., Bhavani R.V. (2022). Agrochemicals, Environment, and Human Health. Annu. Rev. Environ. Resour..

[75] Rathi B.S., Kumar P.S., Show P.-L. (2021). A Review on Effective Removal of Emerging Contaminants from Aquatic Systems: Current Trends and Scope for Further Research. J. Hazard. Mater..

[76] Francis B., Aravindakumar C.T., Brewer P.B., Simon S. (2023). Plant Nutrient Stress Adaptation: A Prospect for Fertilizer Limited Agriculture. Environ. Exp. Bot..

[77] Kumar A., Karn S.K., Hung Y.-T. (2023). Chemicals Used in Agriculture: Hazards and Associated Toxicity Issues: Hazards and Toxicity of Agrochemicals. Waste Treatment in the Biotechnology, Agricultural and Food Industries.

[78] Najam L., Alam T., Aftab T. (2023). Occurrence, Distribution, and Fate of Emerging Persistent Organic Pollutants (POPs) in the Environment. Emerging Contaminants and Plants: Interactions, Adaptations and Remediation Technologies.

[79] Gupta S., Bhadouria R., Tripathi S., Singh P., Singh R., Singh H.P. (2024). Organic Micropollutants in the Environment: Ecotoxicity Potential and Bioremediation Approaches. Organic Micropollutants in Aquatic and Terrestrial Environments.

[80] Jorfi S., Atashi Z., Akhbarizadeh R., Khorasgani Z.N., Ahmadi M. (2019). Distribution and Health Risk Assessment of Organochlorine Pesticides in Agricultural Soils of the Aghili Plain, Southwest Iran. Environ. Earth Sci..

[81] Sharma S., Pathania S., Bhagta S., Kaushal N., Bhardwaj S., Bhatia R.K., Walia A. (2024). Microbial Remediation of Polluted Environment by Using Recombinant *E. coli*: A Review. Biotechnol. Environ..

[82] Sun S., Sidhu V., Rong Y., Zheng Y. (2018). Pesticide Pollution in Agricultural Soils and Sustainable Remediation Methods: A Review. Curr. Pollut. Rep..

[83] Belz R.G., Duke S.O. (2014). Herbicides and Plant Hormesis. Pest Manag. Sci..

[84] Campoy S., Adrio J.L. (2017). Antifungals. Biochem. Pharmacol..

[85] Yadav R., Lahariya V., Vikas, Singh A.K., Das A., Yadav A., Gupta G. (2024). Fluorometric Sensing and Nanomolar Level Detection of Heavy Metal Ions Using Nitrogen Doped Carbon Dots. Emergent Mater..

[86] Aloo B.N. (2024). Pollution of Ground and Surface Waters with Agrochemicals. Handbook of Water Pollution.

[87] Goralczyk K. (2021). A Review of the Impact of Selected Anthropogenic Chemicals from the Group of Endocrine Disruptors on Human Health. Toxics.

[88] Rushing B.R., Selim M.I. (2019). Aflatoxin B1: A Review on Metabolism, Toxicity, Occurrence in Food, Occupational Exposure, and Detoxification Methods. Food Chem. Toxicol..

[89] Bansal A., Sharma M., Pandey A., Shankar J., Singh I., Rajpal V.R., Navi S.S. (2023). Aflatoxins: Occurrence, Biosynthesis Pathway, Management, and Impact on Health. Fungal Resources for Sustainable Economy: Current Status and Future Perspectives.

[90] Gbashi S., Njobeh P.B., Madala N.E., De Boevre M., Kagot V., De Saeger S. (2020). Parallel Validation of a Green-Solvent Extraction Method and Quantitative Estimation of Multi-Mycotoxins in Staple Cereals Using LC-MS/MS. Sci. Rep..

[91] Okechukwu V.O., Adelusi O.A., Kappo A.P., Njobeh P.B., Mamo M.A. (2024). Aflatoxins: Occurrence, Biosynthesis, Mechanism of Action and Effects, Conventional/Emerging Detection Techniques. Food Chem..

[92] Miklós G., Angeli C., Ambrus Á., Nagy A., Kardos V., Zentai A., Kerekes K., Farkas Z., Jóźwiak Á., Bartók T. (2020). Detection of Aflatoxins in Different Matrices and Food-Chain Positions. Front. Microbiol..

[93] IARC (2012). Aflatoxins. IARC Monographs on the Evaluation of Carcinogenic Risks on Humans.

[94] Claeys L., Romano C., De Ruyck K., Wilson H., Fervers B., Korenjak M., Zavadil J., Gunter M.J., De Saeger S., De Boevre M. (2020). Mycotoxin Exposure and Human Cancer Risk: A Systematic Review of Epidemiological Studies. Compr. Rev. Food Sci. Food Saf..

[95] Jaćević V., Dumanović J., Alomar S.Y., Resanović R., Milovanović Z., Nepovimova E., Wu Q., Franca T.C.C., Wu W., Kuča K. (2023). Research Update on Aflatoxins Toxicity, Metabolism, Distribution, and Detection: A Concise Overview. Toxicology.

[96] Schrenk D., Bignami M., Bodin L., Chipman J.K., del Mazo J., Grasl-Kraupp B., Hogstrand C., Hoogenboom L.R., Leblanc J.-C., EFSA Panel on Contaminants in the Food Chain (CONTAM) (2020). Risk Assessment of Aflatoxins in Food. EFSA J..

[97] Omer A.K., Mohammed R.R., Ameen P.S.M., Abas Z.A., Ekici K. (2021). Presence of Biogenic Amines in Food and Their Public Health Implications: A Review. J. Food Prot..

[98] Özogul Y., Özogul F. (2019). Biogenic Amines Formation, Toxicity, Regulations in Food.

[99] Zhang Y., Yu J., Lai S., Song J., Wu X., Wang D., Pang L., Chai T. (2021). Rapid Determination of Histamine Level in Seafood Using Read-out Strips Based on High-Performance Thin Layer Chromatography Modified with Self-Visualization Nanomaterials. Food Control.

[100] U.S. Food and Drug Administration (2012). US Food and Drug Administration Guidelines for the Validation of Chemical Methods for the FDA Foods Program. FdA Foods program Guidelines for Chemical Methods, Version 2012.

[101] Xu X., Liu X., Wang S., Zou Y., Zhang J., Liang L., Wen C., Li Y., Xu X., He X. (2024). Relationship between PAH4 Formation and Thermal Reaction Products in Model Lipids and Possible Pathways of PAHs Formation. J. Hazard. Mater..

[102] Singh L., Agarwal T., Simal-Gandara J. (2020). PAHs, Diet and Cancer Prevention: Cooking Process Driven-Strategies. Trends Food Sci. Technol..

[103] Du W., Jiang S., Lei Y., Wang J., Cui Z., Xiang P., Chang Z., Duan W., Shen G., Qin Y. (2025). Occurrence, Formation Mechanism, and Health Risk of Polycyclic Aromatic Hydrocarbons in Barbecued Food. Ecotoxicol. Environ. Saf..

[104] European Food Safety Authority (EFSA) (2008). Polycyclic Aromatic Hydrocarbons in Food-scientific Opinion of the Panel on Contaminants in the Food Chain. EFSA J..

[105] Singh S., Sivaram N., Dhanjal D.S., Assefa H., Singh J., Ramamurthy P.C. (2024). Navigating the Complexity of Emerging Contaminants: Sources, Impacts, and Remediation Strategies. J. Indian Inst. Sci..

[106] Belay W.Y., Getachew M., Tegegne B.A., Teffera Z.H., Dagne A., Zeleke T.K., Abebe R.B., Gedif A.A., Fenta A., Yirdaw G. (2024). Mechanism of Antibacterial Resistance, Strategies and next-Generation Antimicrobials to Contain Antimicrobial Resistance: A Review. Front. Pharmacol..

[107] Sudarsan J.S., Dogra K., Kumar R., Raval N.P., Leifels M., Mukherjee S., Trivedi M.H., Jain M.S., Zang J., Barceló D. (2024). Tricks and Tracks of Prevalence, Occurrences, Treatment Technologies, and Challenges of Mixtures of Emerging Contaminants in the Environment: With Special Emphasis on Microplastic. J. Contam. Hydrol..

[108] Cechinel M.A.P., Macuvele D.L.P., Padoin N., Riella H.G., Soares C. (2024). Occurrence of Emerging Contaminants in the Environment Causes and Effects. Occurrence, Distribution and Toxic Effects of Emerging Contaminantsx.

[109] Eze C.G., Nwankwo C.E., Dey S., Sundaramurthy S., Okeke E.S. (2024). Food Chain Microplastics Contamination and Impact on Human Health: A Review. Environ. Chem. Lett..

[110] Kumar P., Chaudhary S., Bhalla A. (2024). Occurrence and Fate of Emerging Contaminants with Microplastics Current Scenario, Sources and Effects. Occurrence, Distribution and Toxic Effects of Emerging Contaminantsx.

[111] Molins-Delgado D., Díaz-Cruz M.S., Barceló D., Díaz-Cruz M.S., Barceló D. (2015). Introduction: Personal Care Products in the Aquatic Environment. Personal Care Products in the Aquatic Environment.

[112] Naeem M., Gill R., Gill S.S., Singh K., Sofo A., Tuteja N. (2023). Emerging Contaminants and Their Effect on Agricultural Crops.

[113] Bhavadharini B., Kavimughil M., Malini B., Vallath A., Prajapati H.K., Sunil C.K. (2022). Recent Advances in Biosensors for Detection of Chemical Contaminants in Food—A Review. Food Anal. Methods.

[114] Mirza Alizadeh A., Mohammadi M., Hashempour-baltork F., Hosseini H., Shahidi F. (2025). Process-Induced Toxicants in Food: An Overview on Structures, Formation Pathways, Sensory Properties, Safety and Health Implications. Food Prod. Process. Nutr..

[115] Lee J., Roux S., Le Roux E., Keller S., Rega B., Bonazzi C. (2022). Unravelling Caramelization and Maillard Reactions in Glucose and Glucose + Leucine Model Cakes: Formation and Degradation Kinetics of Precursors, α-Dicarbonyl Intermediates and Furanic Compounds during Baking. Food Chem..

[116] Liu S., Zhou G., Liu H., Han B. (2024). Development Trends in Selective Hydrogenation Upgrading of 5-Hydroxymethylfurfural Catalyzed by Heterogeneous Metal Catalysts. Top. Catal..

[117] Abraham K., Gürtler R., Berg K., Heinemeyer G., Lampen A., Appel K.E. (2011). Toxicology and Risk Assessment of 5-Hydroxymethylfurfural in Food. Mol. Nutr. Food Res..

[118] Czerwonka M., Pietrzak-Sajjad R., and Bobrowska-Korczak B. (2020). Evaluation of 5-Hydroxymethylfurfural Content in Market Milk Products. Food Addit. Contam. Part. A.

[119] Althaiban M.A. (2024). Investigation of Hydroxymethylfurfural Levels in Commercial Acacia Honey for Quality Control: A Systematic Review. Discov. Appl. Sci..

[120] Elbashir A.A., Omar M.M.A., Ibrahim W.A.W., Schmitz O.J., Aboul-Enein H.Y. (2014). Acrylamide Analysis in Food by Liquid Chromatographic and Gas Chromatographic Methods. Crit. Rev. Anal. Chem..

[121] Nematollahi A., Kamankesh M., Hosseini H., Ghasemi J., Hosseini-Esfahani F., Mohammadi A. (2019). Investigation and Determination of Acrylamide in the Main Group of Cereal Products Using Advanced Microextraction Method Coupled with Gas Chromatography-Mass Spectrometry. J. Cereal Sci..

[122] Singh J., Kumar D., Rachamalla M., Jangra A., Prakash C., Kesari K.K., Negi A. (2025). Toxic Effects of Acrylamide and Their Underlying Mechanisms. Sustainable Development Goals Towards Environmental Toxicity and Green Chemistry: Environment and Sustainability.

[123] Song X., Yu J., Yu X., Zhang F., Zeng J., Wan X., Zhang Y. (2025). Cracking the Code of Acrylamide and Nε-(Carboxymethyl)Lysine: Fish Oil Use and Predictive Strategies in Potato Chips during Thermal Processing. Food Chem..

[124] Wu B., Chai X., He A., Huang Z., Chen S., Rao P., Ke L., Xiang L. (2021). Inhibition of Acrylamide Toxicity In Vivo by Arginine-Glucose Maillard Reaction Products. Food Chem. Toxicol..

[125] Cai C., Song Z., Xu X., Yang X., Wei S., Chen F., Dong X., Zhang X., Zhu Y. (2025). The Neurotoxicity of Acrylamide in Ultra-Processed Foods: Interventions of Polysaccharides through the Microbiota–Gut–Brain Axis. Food Funct..

[126] Sebastià A., Fernández-Matarredona C., Castagnini J.M., Barba F.J., Berrada H., Moltó J.C., Pardo O., Esteve-Turrillas F.A., Ferrer E. (2025). Acrylamide Content in Popcorn from Spanish Market: Risk Assessment. Food Chem. Toxicol..

[127] Usman A., Ahmad M. (2016). From BPA to Its Analogues: Is. it a Safe Journey?. Chemosphere.

[128] Kawamura Y., Inoue K., Nakazawa H., Yamada T., Maitani T. (2001). Cause of bisphenol A migration from cans for drinks and assessment of improved cans. Shokuhin Eiseigaku Zasshi.

[129] Noonan G.O., Ackerman L.K., Begley T.H. (2011). Concentration of Bisphenol A in Highly Consumed Canned Foods on the U.S. Market. J. Agric. Food Chem..

[130] Manzoor M.F., Tariq T., Fatima B., Sahar A., Tariq F., Munir S., Khan S., Nawaz Ranjha M.M.A., Sameen A., Zeng X.-A. (2022). An Insight into Bisphenol A, Food Exposure and Its Adverse Effects on Health: A Review. Front. Nutr..

[131] Costa H.E., Cairrao E. (2024). Effect of Bisphenol A on the Neurological System: A Review Update. Arch. Toxicol..

[132] Agarwal A., Gandhi S., Tripathi A.D., Gupta A., Iammarino M., Sidhu J.K. (2025). Food Contamination from Packaging Material with Special Focus on the Bisphenol-A. Crit. Rev. Biotechnol..

[133] EFSA Panel on Food Contact Materials, Enzymes, Flavourings and Processing Aids (CEF) (2015). Scientific Opinion on the Risks to Public Health Related to the Presence of Bisphenol A (BPA) in Foodstuffs. EFSA J..

[134] Deveci G., Tek N.A. (2024). *N*-Nitrosamines: A Potential Hazard in Processed Meat Products. J. Sci. Food Agric..

[135] Wang W., Song Z., Jing Y., Wei X., Li H., Xie J., Shen M. (2025). Formation of Advanced Glycation End-Products and *N*-Nitrosamines in Salami of Different Recipes and Fermented at Different Stages. Food Chem..

[136] Wang Y., Liu Y., Huang X., Xiao Z., Yang Y., Yu Q., Chen S., He L., Liu A., Liu S. (2023). A Review on Mechanistic Overview on the Formation of Toxic Substances during the Traditional Fermented Food Processing. Food Rev. Int..

[137] Zhou F., Liao X., Li L., Huang Y., Qi H., Li Q., Zou J., Zhou Z., Tu F., Wei M. (2025). Laws and Mechanisms of Inorganic Ammonia Mediates Organic NDMA Formation during Ozonation of Amines. Sep. Purif. Technol..

[138] Paustenbach D.J., Brown S.E., Heywood J.J., Donnell M.T., Eaton D.L. (2024). Risk Characterization of *N*-Nitrosodimethylamine in Pharmaceuticals. Food Chem. Toxicol..

[139] Tayel D.I., Farrag N.K., Aborhyem S.M. (2025). Dietary Intake and Risk Assessment of Nitrosamine in Processed Meat Products among Medical Staff during Their Night Shift. Sci. Rep..

[140] Ramezani H., Hosseini H., Kamankesh M., Ghasemzadeh-Mohammadi V., Mohammadi A. (2015). Rapid Determination of Nitrosamines in Sausage and Salami Using Microwave-Assisted Extraction and Dispersive Liquid–Liquid Microextraction Followed by Gas Chromatography–Mass Spectrometry. Eur. Food Res. Technol..

[141] Devi P., Saini S., Kim K.-H. (2019). The Advanced Role of Carbon Quantum Dots in Nanomedical Applications. Biosens. Bioelectron..

[142] Ji C., Zhou Y., Leblanc R.M., Peng Z. (2020). Recent Developments of Carbon Dots in Biosensing: A Review. ACS Sens..

[143] Sharma A., Gadly T., Gupta A., Ballal A., Ghosh S.K., Kumbhakar M. (2016). Origin of Excitation Dependent Fluorescence in Carbon Nanodots. J. Phys. Chem. Lett..

[144] Zhang Z., Chen J., Duan Y., Liu W., Li D., Yan Z., Yang K. (2018). Highly Luminescent Nitrogen-Doped Carbon Dots for Simultaneous Determination of Chlortetracycline and Sulfasalazine. Luminescence.

[145] Li M., Chen T., Gooding J.J., Liu J. (2019). Review of Carbon and Graphene Quantum Dots for Sensing. ACS Sens..

[146] Chobpattana V., Sangtawesin T., Khaopueak P., Wechakorn K. (2025). Sugar Derived-Fluorescent Carbon Quantum Dots Conjugated Glutathione for Sensing Heavy Metal Ions and Antioxidant Activity. Mater. Sci. Eng. B.

[147] Arvapalli D.M., Sheardy A.T., Alapati K.C., Wei J. (2020). High Quantum Yield Fluorescent Carbon Nanodots for Detection of Fe (III) Ions and Electrochemical Study of Quenching Mechanism. Talanta.

[148] Wahyudi S., Rizoputra I., Panatarani C., Faizal F., Bahtiar A. (2024). Green Synthesis of Carbon Nanodots (CNDs) Moderated by Flavonoid Extracts from Moringa Oleifera Leaves and Co-Doped Sulfur/Nitrogen (NS–CNDs–Fla) and Their Potential for Heavy Metals Sensing Application. J. Fluoresc..

[149] Kim Y., Jeon Y., Na M., Hwang S.-J., Yoon Y. (2024). Recent Trends in Chemical Sensors for Detecting Toxic Materials. Sensors.

[150] Zhao L., Zhang M., Mujumdar A.S., Wang H. (2023). Application of Carbon Dots in Food Preservation: A Critical Review for Packaging Enhancers and Food Preservatives. Crit. Rev. Food Sci. Nutr..

[151] Zhao D., Xu X., Wang X., Xu B., Zhang F., Wu W. (2023). Synthesis of a Core–Shell Magnetic Covalent Organic Framework for the Enrichment and Detection of Aflatoxin in Food Using HPLC-MS/MS. Microchim. Acta.

[152] Alsammarraie F.K., Lin M. (2017). Using Standing Gold Nanorod Arrays as Surface-Enhanced Raman Spectroscopy (SERS) Substrates for Detection of Carbaryl Residues in Fruit Juice and Milk. J. Agric. Food Chem..

[153] Goswami N., Yao Q., Luo Z., Li J., Chen T., Xie J. (2016). Luminescent Metal Nanoclusters with Aggregation-Induced Emission. J. Phys. Chem. Lett..

[154] Buzuk M. (2024). Chemical Sensors for Toxic Chemical Detection. Sensors.

[155] de Medeiros T.V., Manioudakis J., Noun F., Macairan J.-R., Victoria F., Naccache R. (2019). Microwave-Assisted Synthesis of Carbon Dots and Their Applications. J. Mater. Chem. C.

[156] Nguyen D.H.H., El-Ramady H., Prokisch J. (2025). Food Safety Aspects of Carbon Dots: A Review. Environ. Chem. Lett..

[157] Li G., Liu C., Zhang X., Luo P., Lin G., Jiang W. (2021). Highly Photoluminescent Carbon Dots-Based Immunosensors for Ultrasensitive Detection of Aflatoxin M1 Residues in Milk. Food Chem..

[158] Singh H., Singh S., Bhardwaj S.K., Kaur G., Khatri M., Deep A., Bhardwaj N. (2022). Development of Carbon Quantum Dot-Based Lateral Flow Immunoassay for Sensitive Detection of Aflatoxin M1 in Milk. Food Chem..

[159] Rai M., Ingle A.P., Törős G., Prokisch J. (2024). Assessing the Efficacy of Carbon Nanodots Derived from Curcumin on Infectious Diseases. Expert Rev. Anti Infect. Ther..

[160] Kaur H., Bhattu M., Chakroborty S., Aulakh M.K., Mutreja V., Verma M., Tiwari K., Chakraborty C., Darwish I.A. (2025). Highly Green Fluorescent Carbon Dots from Gallic Acid: A Turn-On Sensor toward Pb^2+^ Ions. ACS Omega.

[161] Sharma N., Thakur S., Bains A., Goksen G., Ali N., Ansari M.A., Kopsacheili A., Proestos C., Chawla P. (2025). Green Hydrothermal Approach for the Synthesis of Carbon Quantum Dots from Waste Tea Bags for Acrylamide Detection in Drinking Water: A Fluorescence Assay Validated by HPLC-PDA Analysis. Food Chem. X.

[162] Zhang X., Wang J., Hasan E., Sun X., Asif M., Aziz A., Lu W., Dong C., Shuang S. (2024). Bridging Biological and Food Monitoring: A Colorimetric and Fluorescent Dual-Mode Sensor Based on N-Doped Carbon Dots for Detection of pH and Histamine. J. Hazard. Mater..

[163] Cao C., Guo W. (2024). Carbon Dots-Based Fluorescent Probe for the Detection of Imidacloprid Residue in Leafy Vegetables. Food Chem..

[164] Durrani S., Zhang J., Durrani F., Wang Z., Mukramin, Xu K.-F., Wang H., Khan H., Wu F.-G., Lin F. (2025). Triple Channel Fluorescence Na-Ca-Cl-Doped Carbon Dots for Erythrosine Detection in Food Samples and Living Cells. J. Mol. Struct..

[165] Li N., Liu T., Liu S.G., Lin S.M., Fan Y.Z., Luo H.Q., Li N.B. (2017). Visible and Fluorescent Detection of Melamine in Raw Milk with One-Step Synthesized Silver Nanoparticles Using Carbon Dots as the Reductant and Stabilizer. Sens. Actuators B Chem..

[166] Zhuang Q., Li L., Ding Y., Zeng H., Wu Y. (2019). Highly Luminescent Nitrogen-Doped Carbon Dots as “Turn-On” Fluorescence Probe for Selective Detection of Melamine. ChemistrySelect.

[167] Waluyo R., Manopo J., Isnaeni, Darma Y. (2025). Unraveling Excitation-Dependent Fluorescence of Nitrogen and Sodium Co-Doped Carbon Dots for Dual Detection of Fe^3+^ and Ag^+^. Colloids Surf. A Physicochem. Eng. Asp..

[168] Zhao X., Li Q., Li H., Wang Y., Xiao F., Yang D., Xia Q., Yang Y. (2023). SERS Detection of Hg^2+^ and Aflatoxin B1 through on–off Strategy of Oxidase-like Au@HgNPs/Carbon Dots. Food Chem..

[169] Aafria S., Sharma M. (2025). Development of a Rapid and Ultrasensitive Acrylamide Nanosensor Based on TiO2 NPs/GQDs Nanocomposite. J. Food Compos. Anal..

[170] Alhazzani K., Alanazi A.Z., Ibrahim H., Mostafa A.M., Barker J., Mahmoud A.M., El-Wekil M.M., Ali A.-M.B.H. (2025). L-Asparaginase-Mediated pH Shift and Carbon Dot Fluorescence Modulation: A Sensitive Ratiometric Method for Quantifying L-Asparagine in Diverse Potato Varieties under Variable Storage Conditions. Food Chem..

[171] Medhi M., Yumnam M., Mudoi P., Mishra P. (2025). Green Florescent Carbon Dots Synthesized from Various Household Green Wastes for Detection of Parathion Methyl Pesticide. J. Lumin..

[172] Wang Y., Wang K., Wang M., Shan R., Li X., Zhang H. (2025). Novel Ratiometric Fluorescence Sensor for Organophosphorus Pesticides via Carbon Dots Supported Zirconium-Based Metal Organic Frameworks. Food Control.

[173] Rahmah A.S., Heryanto H., Rinovian A., Yudasari N., Tahir D. (2025). Structural Characterization of Carbon Quantum Dots Derived from Tea Residue and Their Photocatalytic Application in CQDs-Modified Al_2_(SO_4_)_3_ Nanoparticles for Sustainable Pesticide Degradation. Mater. Chem. Phys..

[174] Renu, Nidhi, Kaur P., Komal, Minakshi, Paulik C., Kaushik A., Singhal S. (2025). Rational Design of *Boerhavia Diffusa* Derived CoFe_2_O_4_-Carbon dots@Boehmite Platform for Photocatalysis and Ultra Trace Monitoring of Hazardous Pesticide and UO_2_^2+^ Ions. Spectrochim. Acta Part A Mol. Biomol. Spectrosc..

[175] Kaur H., Jain V., Renuka Jyothi S., Bhanot D., Kumari B., Verma M., Mutreja V., Bhattu M. (2025). Dual-Emission Carbon Dots from Gallic Acid for Selective Turn off Fluorescent Detection of Chlorpyrifos in Wastewater. J. Mol. Struct..

[176] Vadia F.Y., Jha S., Mehta V.N., Park T.J., Malek N.I., Kailasa S.K. (2025). Development of Sustainable Fluorescence Approach with Red Emissive Carbon Dots Derived from *Grewia Asiatica* Fruit for the Detection of Quinalphos. J. Photochem. Photobiol. A Chem..

[177] Kumar V., Kaushal I., Sharma A.K., Sharma S., Kumar A., Bhukal S., Rani J., Saharan P. (2025). Biowaste-Derived Multifunctional CeO_2_@carbon Dot Nanospheres for Efficient Sono-Catalytic Degradation of Rhodamine B Dye and Electrochemical Sensing of 4-Nitrophenol. Appl. Surf. Sci..

[178] Pan M., Gao M., Cui J., Gao R., Li H., Sun J., Chen W., Wang S. (2025). Fluorescent Molecularly Imprinted Hydrogel Sensing Strip Based on Nitrogen-Doped Carbon Dots and Inverse Opal Photonic Crystals Applying for Effective Detection for Imidacloprid in Fruits and Vegetables. Food Chem..

[179] Mallik A., Hazra M., Adak M.K., Nag R., Pandey A., Sahoo G.P. (2025). Fluorescent Probe Based on Boron-nitrogen Co-Doped Carbon Dots for the Rapid Detection of Acephate Residue in Vegetables and Water. Diam. Relat. Mater..

[180] Muangmora R., Rojviroon O., Kemacheevakul P., Chuangchote S., Rajendran R., Phouheuanghong P., Arumugam P., Paramasivam S., Rojviroon T. (2025). Spent Coffee Ground-Derived Carbon Quantum Dot Composite with Metal Oxides for Photocatalytic Degradation of Carbaryl in Water and Antibacterial Application. J. Water Process Eng..

[181] Silaram W., Boonlerd R., Rattanaphonsaen P., Khiaophong W., Kachangoon R., Teshima N., Pakkethati K., Mukdasai S., Ponhong K., Vichapong J. (2025). Functional Graphene Carbon Dots in Corn Stalk Pith as an Efficient Sorbent for Preconcentration and Trace Determination of Triazole Residues in Rice Samples. Microchem. J..

[182] Zhang J., Chu S., Tao C., Yan J., Jiang Y., Lu Y. (2024). Nitrogen-Doped Carbon Dots as Efficient Turn-on Fluorescent Probe for Assay of Organophosphorus Pesticides. ChemPhysMater.

[183] Liu H., Wu S. (2025). Blue Fluorescent Carbon Dots Doped with Nitrogen and Sulfur as a Dual-Functional Fluorescent Probe for the Detection of Hg^2+^ and Chloramphenicol. J. Mol. Struct..

[184] Tran N.B., Nguyen Q.K., Ngoc Dang T.M., Tran T.D., Nguyen T.M., Thuong Nguyen T.K., Vu D.T., Pham B., Huong Nguyen T.A., Mai Pham T.N. (2025). Nitrogen Doped Carbon Quantum Dots (N-CQDs) Synthesized by a Microwave Assisted Hydrothermal Method in Combination with Gold Nanoparticles (AuNPs) as Sensitive Dual Sensor for Antibiotic Meropenem Detection. Microchem. J..

[185] Xia W., Wu Z., Hou B., Cheng Z., Bi D., Chen L., Chen W., Yuan H., Koole L.H., Qi L. (2025). Inactivation of Antibiotic Resistant Bacteria by Nitrogen-Doped Carbon Quantum Dots through Spontaneous Generation of Intracellular and Extracellular Reactive Oxygen Species. Mater. Today Bio.

[186] Miao H., Wang P., Wu J., Li X., Du Y., Yan H., You Q., Dong W., Li L. (2025). Highly Efficient and Broad-Spectrum Antibacterial Carbon Dots Combat Antibiotic Resistance. Talanta.

[187] Ebere Enyoh C., Wang Q. (2025). Box–Behnken Design and Machine Learning Optimization of PET Fluorescent Carbon Quantum Dots for Removing Fluoxetine and Ciprofloxacin with Molecular Dynamics and Docking Studies as Potential Antidepressant and Antibiotic. Sep. Purif. Technol..

[188] Wang F., Li X., Zhang Y., Li H., Jiang S., Han J., Gong W., Li D., Yao Z. (2025). A Ratiometric Fluorescent Probe for the Determination of Quinolone Antibiotics in Milk Based on N and S Co-Doped Carbon Quantum Dots. Food Control.

[189] Karthikeyan M., Hemalatha M., Venkatasubbu D.G., Abimanyu S., Aravind M., Rathinsabapathi P. (2025). Label-Free Detection of Ampicillin in Milk and Water Using Fluorescence Carbon Dots Synthesised from *Citrus Limetta* Peel. Diam. Relat. Mater..

[190] Du J., Liu S., Hou J., Wu X., Si H., Guo X., Zhuo S. (2025). Curcumin-Derived Water-Soluble Carbon Dots without Detectable Resistance: Dual Potentials for Antimicrobial Activity and Infected Wound Healing. Diam. Relat. Mater..

[191] Hu J., Long X., Wu S. (2025). High-Performance Nitrogen-Doped Green Fluorescent Carbon Dots for Applications in Rapid Detection of Chlortetracycline and Fluorescent Film. Spectrochim. Acta Part A Mol. Biomol. Spectrosc..

[192] Hu J., Ma Y., Wu S. (2025). Synthesis and Application of Optically Stable Red Fluorescent Carbon Dots for Sensitive and Selective Detection of Ceftazidime. Spectrochim. Acta Part A Mol. Biomol. Spectrosc..

[193] Shen S., Qi H., Yi T., Jing T., Li J., Gao Y., Zeng Q., Zhao H. (2025). Quaternary Ammonium Salt-Derived Carbon Dots for Antibacterial Efficacy and Tetracycline Sensing. J. Mol. Struct..

[194] Luo Y., Zhang H., Liu J., Jamil A., Hou Y., Li Q., Zhao M., Zhao C., Li W., Hong B. (2025). Deep Eutectic Solvent-Based Magnetic Molecular Nanomaterials Coupled with Fluorescent Carbon Dots as a New Strategy to Highly Enrich and Sensitively Detect Doxycycline in Food Matrices. Food Chem. X.

[195] Buzid A., Luong J.H.T., Azad U.P., Chandra P. (2023). Electrochemical Sensing and Biosensing-Based on Carbon Nanodots. Handbook of Nanobioelectrochemistry: Application in Devices and Biomolecular Sensing.

[196] Mei Y., He C., Zeng W., Luo Y., Liu C., Yang M., Kuang Y., Lin X., Huang Q. (2022). Electrochemical Biosensors for Foodborne Pathogens Detection Based on Carbon Nanomaterials: Recent Advances and Challenges. Food Bioprocess Technol..

[197] Gauglitz G. (2018). Analytical Evaluation of Sensor Measurements. Anal. Bioanal. Chem..

[198] Rajoriya K., Pratibha, Abhijeet, Meena R., Kumari A. (2024). Synthesis of Fluorometric Carbon Nano Dots(CNDs) for Selective Sensing of Biologically Important Fe^3+^ and Cu^2+^ Metal Ions and Evaluating Their Antioxidant Capacity. J. Fluoresc..

[199] Zito C.A., Orlandi M.O., Volanti D.P. (2018). Accelerated Microwave-Assisted Hydrothermal/Solvothermal Processing: Fundamentals, Morphologies, and Applications. J. Electroceram.

[200] Khan S., Dunphy A., Anike M.S., Belperain S., Patel K., Chiu N.H.L., Jia Z. (2021). Recent Advances in Carbon Nanodots: A Promising Nanomaterial for Biomedical Applications. Int. J. Mol. Sci..

[201] Yan F., Jiang Y., Sun X., Bai Z., Zhang Y., Zhou X. (2018). Surface Modification and Chemical Functionalization of Carbon Dots: A Review. Microchim. Acta.

[202] Qi X., Jin W., Tang C., Xiao X., Li R., Ma Y., Ma L. (2025). pH Monitoring in High Ionic Concentration Environments: Performance Study of Graphene-Based Sensors. Anal. Sci..

[203] Sankhla L., Kushwaha H.S. (2024). Development of an Opto-Electrochemical Sensor for the Detection of Malathion Using Manganese Metal–Organic Framework (Mn-MOF). J. Mater. Sci. Mater. Eng..

[204] Yoo H., Jo H., Soo Oh S. (2020). Detection and beyond: Challenges and Advances in Aptamer-Based Biosensors. Mater. Adv..

[205] Chen M., Li H., Xue X., Tan F., Ye L. (2024). Signal Amplification in Molecular Sensing by Imprinted Polymers. Microchim. Acta.

[206] Gonçalves R.G.L., Lopes P.A., Pochapski D.J., de Oliveira L.C.A., Pinto F.G., Neto J.L., Tronto J. (2022). Effect of pH, Ionic Strength, and Temperature on the Adsorption Behavior of Acid Blue 113 onto Mesoporous Carbon. Environ. Sci. Pollut. Res..

[207] Saputra H.A. (2023). Electrochemical Sensors: Basic Principles, Engineering, and State of the Art. Monatsh Chem..

[208] He X., Liu Z., Shen G., He X., Liang J., Zhong Y., Liang T., He J., Xin Y., Zhang C. (2021). Microstructured Capacitive Sensor with Broad Detection Range and Long-Term Stability for Human Activity Detection. npj Flex. Electron..

[209] Yaroshenko I., Kirsanov D., Marjanovic M., Lieberzeit P.A., Korostynska O., Mason A., Frau I., Legin A. (2020). Real-Time Water Quality Monitoring with Chemical Sensors. Sensors.

[210] Pan Y., Zhang J., Guo X., Li Y., Li L., Pan L. (2024). Recent Advances in Conductive Polymers-Based Electrochemical Sensors for Biomedical and Environmental Applications. Polymers.

[211] Boonruang S., Naksen P., Anutrasakda W., Chansaenpak K., Promarak V., Saenmuangchin R., Phechkrajang C., Jarujamrus P. (2021). Use of Nitrogen-Doped Amorphous Carbon Nanodots (N-CNDs) as a Fluorometric Paper-Based Sensor: A New Approach for Sensitive Determination of Lead(II) at a Trace Level in Highly Ionic Matrices. Anal. Methods.

[212] Vaks J.E., Hemyari P., Rullkoetter M., Santulli M.J., Schoenbrunner N. (2016). Verification of Claimed Limit of Detection in Molecular Diagnostics. J. Appl. Lab. Med..

[213] Chauhan D.S., Quraishi M.A., Verma C. (2022). Carbon Nanodots: Recent Advances in Synthesis and Applications. Carbon Lett..

[214] Hnasko R.M., Hnasko R. (2015). Bioconjugation of Antibodies to Horseradish Peroxidase (HRP). ELISA: Methods and Protocols.

[215] Nabi Duman A., Jalilov A.S. (2024). Machine Learning for Carbon Dot Synthesis and Applications. Mater. Adv..

[216] Singh P., Arpita, Kumar S., Kumar P., Kataria N., Bhankar V., Kumar K., Kumar R., Hsieh C.-T., Shiong Khoo K. (2023). Assessment of Biomass-Derived Carbon Dots as Highly Sensitive and Selective Templates for the Sensing of Hazardous Ions. Nanoscale.

[217] Gao Y., Wang Q., Ji G., Li A., Niu J. (2021). Doping Strategy, Properties and Application of Heteroatom-Doped Ordered Mesoporous Carbon. RSC Adv..

[218] Zou Z., Ji Y., Schwaneberg U. (2024). Empowering Site-Specific Bioconjugations In Vitro and In Vivo: Advances in Sortase Engineering and Sortase-Mediated Ligation. Angew. Chem. Int. Ed..

[219] Hu Y., Seivert O., Tang Y., Karahan H.E., Bianco A. (2024). Carbon Dot Synthesis and Purification: Trends, Challenges and Recommendations. Angew. Chem. Int. Ed..

[220] Chiu Y.H., Guo Y.-T., Rinawati M., Chang C.-C., Chang L.-Y., Yeh M.-H. (2024). Developing Flexible Carbon Nitride Quantum Dots Decorated Polyaniline Nanocomposite Layer for a Non-Invasive Wearable Sweat Biosensor for Glucose Monitoring. ECS Meet. Abstr..

[221] Liu C., Lin X., Liao J., Yang M., Jiang M., Huang Y., Du Z., Chen L., Fan S., Huang Q. (2024). Carbon Dots-Based Dopamine Sensors: Recent Advances and Challenges. Chin. Chem. Lett..

[222] Sun F., Dong G., Jiang F., Wang X., Diao B., Li X., Joo S.W., Zhang L., Kim S.H., Cong C. (2024). Carbon Quantum Dot/Multiwalled Carbon Nanotube-Based Self-Powered Strain Sensors for Remote Human Motion Detection. ACS Appl. Nano Mater..

[223] Sengupta J., Hussain C.M. (2022). Decadal Journey of CNT-Based Analytical Biosensing Platforms in the Detection of Human Viruses. Nanomaterials.

[224] Banerjee S.L., Tirrell M.V. (2023). Carbon Nanotube-Based Chemical Sensors. Materials for Chemical Sensors.

[225] Munawar A., Hussain Khan R.F., Iqbal M.Z., Rehman A., Bajwa S.Z., Khan W.S., Chaudhry M.A., Hussain R., Butt F.K. (2022). 18−Drug-Detection Performance of Carbon Nanotubes Decorated with Metal Oxide Nanoparticles. Metal Oxide-Carbon Hybrid Materials.

[226] Meskher H., Mustansar H.C., Thakur A.K., Sathyamurthy R., Lynch I., Singh P., Han T.K., Saidur R. (2023). Recent Trends in Carbon Nanotube (CNT)-Based Biosensors for the Fast and Sensitive Detection of Human Viruses: A Critical Review. Nanoscale Adv..

